# Long-range Order in Canary Song

**DOI:** 10.1371/journal.pcbi.1003052

**Published:** 2013-05-02

**Authors:** Jeffrey E. Markowitz, Elizabeth Ivie, Laura Kligler, Timothy J. Gardner

**Affiliations:** 1 Department of Cognitive and Neural Systems, Boston University, Boston, Massachusetts, United States of America; 2 Center of Excellence for Learning in Education, Science and Technology, Boston, Massachusetts, United States of America; 3 Department of Biology, Boston University, Boston, Massachusetts, United States of America; The Pennsylvania State University, United States of America

## Abstract

Bird songs range in form from the simple notes of a Chipping Sparrow to the rich performance of the nightingale. Non-adjacent correlations can be found in the syntax of some birdsongs, indicating that the choice of what to sing next is determined not only by the current syllable, but also by previous syllables sung. Here we examine the song of the domesticated canary, a complex singer whose song consists of syllables, grouped into phrases that are arranged in flexible sequences. Phrases are defined by a fundamental time-scale that is independent of the underlying syllable duration. We show that the ordering of phrases is governed by long-range rules: the choice of what phrase to sing next in a given context depends on the history of the song, and for some syllables, highly specific rules produce correlations in song over timescales of up to ten seconds. The neural basis of these long-range correlations may provide insight into how complex behaviors are assembled from more elementary, stereotyped modules.

## Author Summary

Bird songs range in form from the simple notes of a Chipping Sparrow to the complex repertoire of the nightingale. Recent studies suggest that bird songs may contain non-adjacent dependencies where the choice of what to sing next depends on the history of what has already been produced. However, the complexity of these rules has not been examined statistically for the most elaborate avian singers. Here we show that one complex singer—the domesticated canary—produces a song that is strongly influenced by long-range rules. The choice of how long to repeat a given note or which note to choose next depends on the history of the song, and these dependencies span intervals of time much longer than previously assumed for birdsong. Like most forms of human music, the songs of canaries contain patterns expressed over long timescales, governed by rules that apply to multiple levels of a temporal hierarchy. This vocal complexity provides a valuable model to examine how ordered behaviors are assembled from more elementary neural components in a relatively simple neural circuit.

## Introduction

Brains build complex behaviors from simple modules [1], [2].The ultimate example is speech where sequences of phonemes form words that in turn are rearranged to form sentences. So too, the complex performances of a musician or swordfighter are composed of discrete motor gestures that may be composed of more elementary motor modules or muscle synergies [3]. Songbirds, in their own ways, build complex vocal forms from elementary units known as syllables. Among the 4500+ species of songbirds, simple and complex songs can be found, and a rich history of detailed song descriptions can be found across a wide variety of literature [4]–[12]. However, quantitative information about the statistical complexity of song is available only for a few species [4], [8]–[12]. Birdsong has often been described in terms of first-order transition statistics, e.g. between adjacent syllables [13] in the zebra finch or syllable chunks [9], [14] in the nightingale and Bengalese finch. However, analysis of the Bengalese finch song also reveals non-adjacent dependencies where transition probabilities between syllables depend not only on the current active syllable, but also one or more prior syllables sung [15]–[17]. Formally, this implies that song syntax must be modeled with a second-order or higher order Markov chain. Higher-order Markov chains can also be represented through first-order statistics in a hidden Markov model (HMM). In the latter case, statistically complex sequences will require a large number of hidden states, relative to the number of observed syllables.

In addition to the detailed quantitative studies of syntax in Bengalese finches in laboratory settings, many field studies have described an array of influences on the delivery of song. For example, some species such as the swamp sparrow engage in antiphonal song type-matching—selecting a song that best matches what the neighbor just sang [18]. In this case, an auditory stimulus is involved in the selection of elements from a vocal repertoire, and the choices are not simply determined by the current motor state [19]. Other examples of complex vocal behavior can be found for species that sing many song types. In some cases, e.g. with Western Meadowlarks and American Redstarts, the probability of producing a given song type decreases after the first time it is delivered in a bout of singing, and as a result, the full repertoire of songs is expressed more frequently than expected if the selection of songs was random [4], [12]. In a related example of song performance memory, nightingales can pause for a few seconds, and then resume singing where they left off in a ordered set of songs [20]. Taken together, numerous threads suggest that songbirds can maintain a memory trace for songs recently heard or sung for at least a few seconds. If syllables sung or heard can introduce a memory trace that lasts for seconds, and if this memory trace can impact future decisions about what to sing, then a substrate exists that could introduce long-range correlations between decision points in song. How deep is the memory for past choices in song among the most elaborate singers?

One of the most complex singers that can be easily reared in a laboratory setting is the domesticated canary. Here we investigated the long, complex songs of the Belgian Waterslager strain. We show that their songs are governed by long-range correlations in syntax at multiple hierarchical levels. The time a bird spends repeating a syllable or the choice of what to sing next depends on the history of the song and correlations between the past and present can extend over durations up to 10 seconds, encompassing 4 or more phrases consisting of dozens of syllable repeats. Canary song, like most popular music, contains structure in a range of time-scales through which sequence flexibility is balanced by long-range order [21]. The neural basis of these long-range correlations may provide insight into how complex behaviors are assembled from more elementary, stereotyped behavioral modules.

## Results

### Phrase time-scales

**Figure 1:**
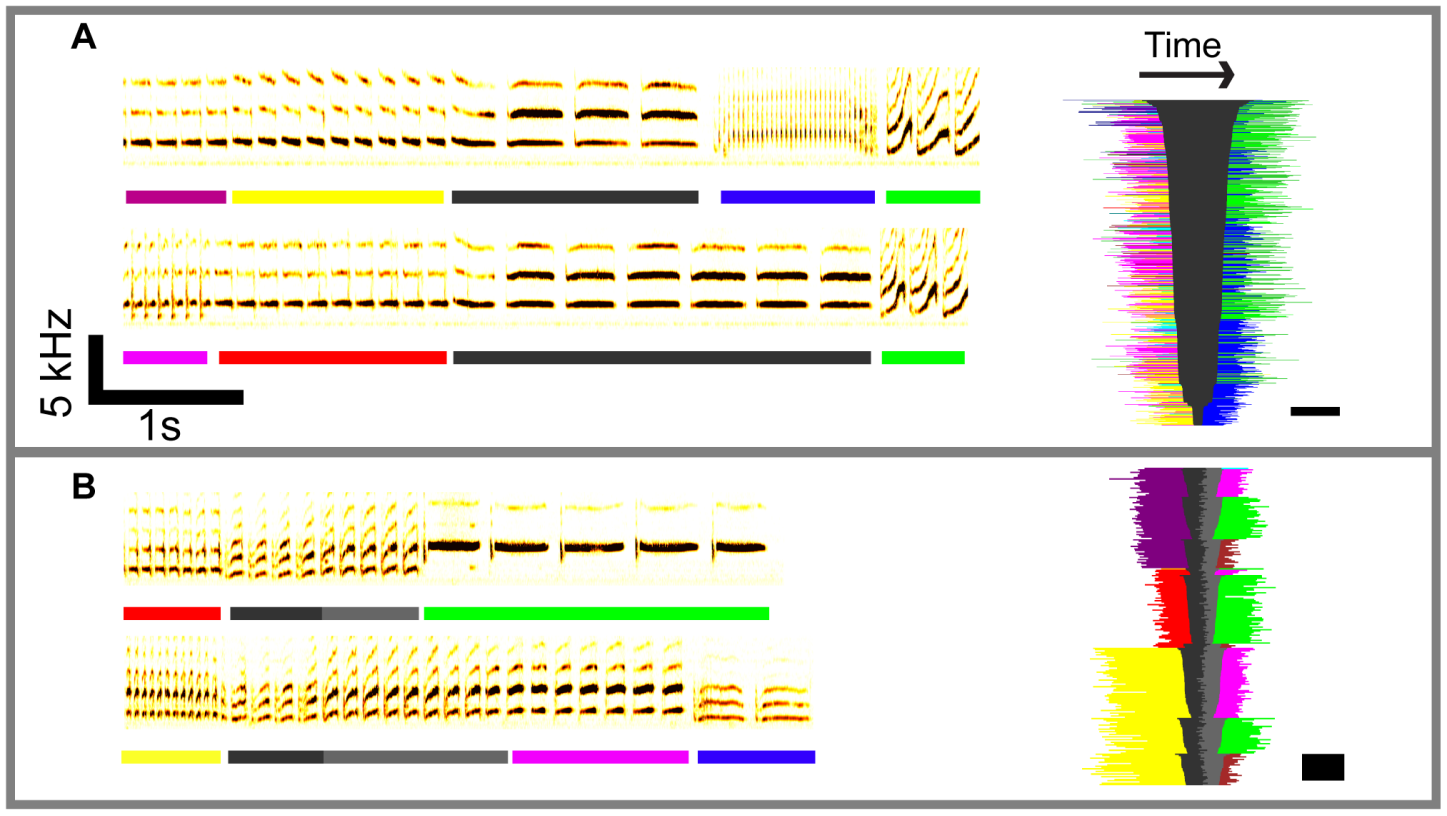
Sonograms show a relationship between phrase duration, the context of a phrase, and future choices. Phrases consist of repetitions of elementary units—the syllables. Distinct phrase types are indicated by colored bars beneath each sonogram. On the right side of each sonogram is a “barcode” summary of all occurrences of the phrases shown in the sonograms. The phrases before (left) and after (right) the gray phrase are color-coded by syllable identity, and the length of the bars in each row indicates duration of the phrase. A square flanks each barcode to indicate the scale, with the width corresponding to 2 seconds and the height to 20 trials. **A**, A case where the duration of a phrase predicts the future path—the barcode is sorted by duration of the black phrase. Short black bars on the bottom of the barcode typically lead to blue, while long black bars at the top typically lead to green. **B**, A case where the identity of the starting phrase (red, yellow, or purple) determines which phrase type comes after the black/gray phrase (green or magenta for example.) The barcode in this panel is sorted by the phrase that comes before the black/gray phrase.

The smallest indivisible unit of the domesticated canary's song is the syllable, a stereotyped sound that typically ranges from 20 to 200 ms in duration. The adult repertoire usually contains between 25–35 distinct syllable types whose acoustic forms are learned by an interplay between innate programming and flexible imitation [22]. Syllables are repeated multiple times to form a phrase, which can range from 500 ms to 3 s, and phrases are flexibly chained together to form songs (**Fig. 1**), typically 5–15 s long [23]. In the present context, the term “phrase type” refers to the syllable type repeated in a given phrase.

**Figure 2:**
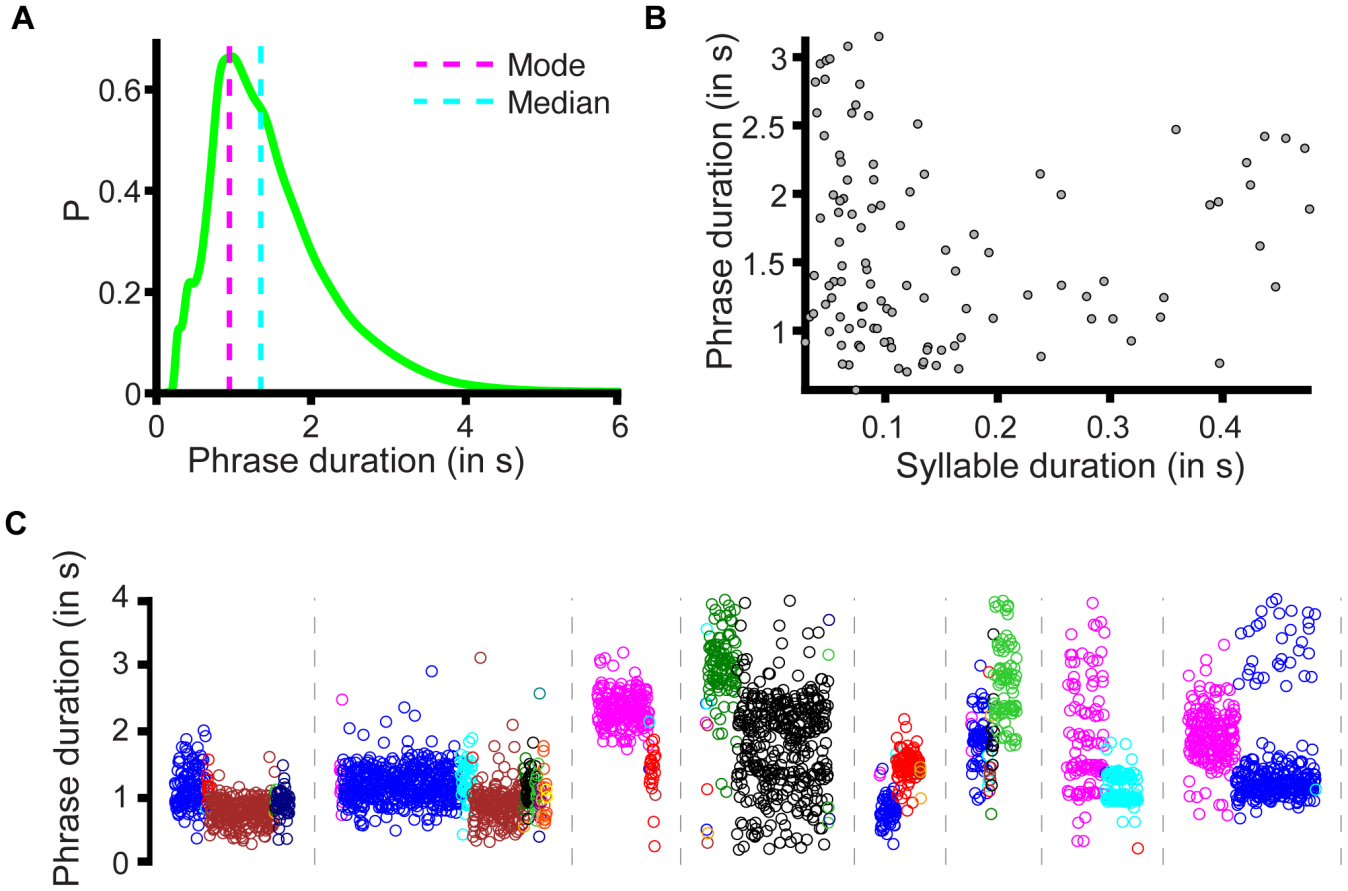
Phrase length is defined by a typical time-scale and depends on context. **A,** Probability density of phrase durations for all 6 birds (mode = .9416, .8132–1.407 95% bootstrap confidence interval, median = 1.3482, 1.3375–1.3589). **B,** A scatter plot of phrase duration plotted against syllable duration for 6 birds reveals no general correlation between phrase length and syllable length (r = .013,p = .90). **C,** Examples of the effect of phrase context on phrase duration. Each group of points (separated by dotted lines) indicates the duration of a different phrase type, while the colors (arbitrary) indicate different preceding phrase types. (These examples draw from all 6 birds, all phrase durations are given in **Fig. S12**).

These two fundamental units of the canary song—syllables and phrases—form distinct time-scales. Syllable durations range from 28 to 480 milliseconds, while phrase time-scales are on the order of a second (1.3375–1.3589 95% bootstrap confidence interval of the median), and the full song is an order of magnitude longer (**Fig. 1** and **Fig. 2**). **Fig. 2b** shows that there is no general correlation between syllable duration and phrase duration (r = .013, p = .90); canaries persist on a single syllable for a duration that is roughly one second, whether the syllable is short or long [24]. To produce a phrase of the characteristic duration, the shortest syllables are repeated 20–30 times, while the longest syllables are repeated only 3–4 times. We discuss later the implications of a phrase time-scale that is not simply related to syllable time-scales.

### Correlations between phrase durations and syllable choices

As a group, there is no general correlation between phrase length and syllable length (**Fig. 2b**). However, particular syllable types do have their own characteristic phrase lengths, and the duration of specific phrases can vary depending on the context in which they occur. Specifically, we found highly significant mutual dependence between the length of specific phrases and the phrase type sung *before* or *after* the phrase (**Fig. 1a** and **Fig. S1**) (81 phrase types examined in 6 birds, p<.001 Fisher-Freeman-Halton test, see **Materials and Methods**). For a given phrase type, the length of a phrase can depend on the recent history of the song. Moreover, the choice of what to sing at a branch-point can depend not only on the currently active syllable, but also on the amount of time elapsed since the onset of the last phrase.

### Syllable stability across the phrase

**Figure 3:**
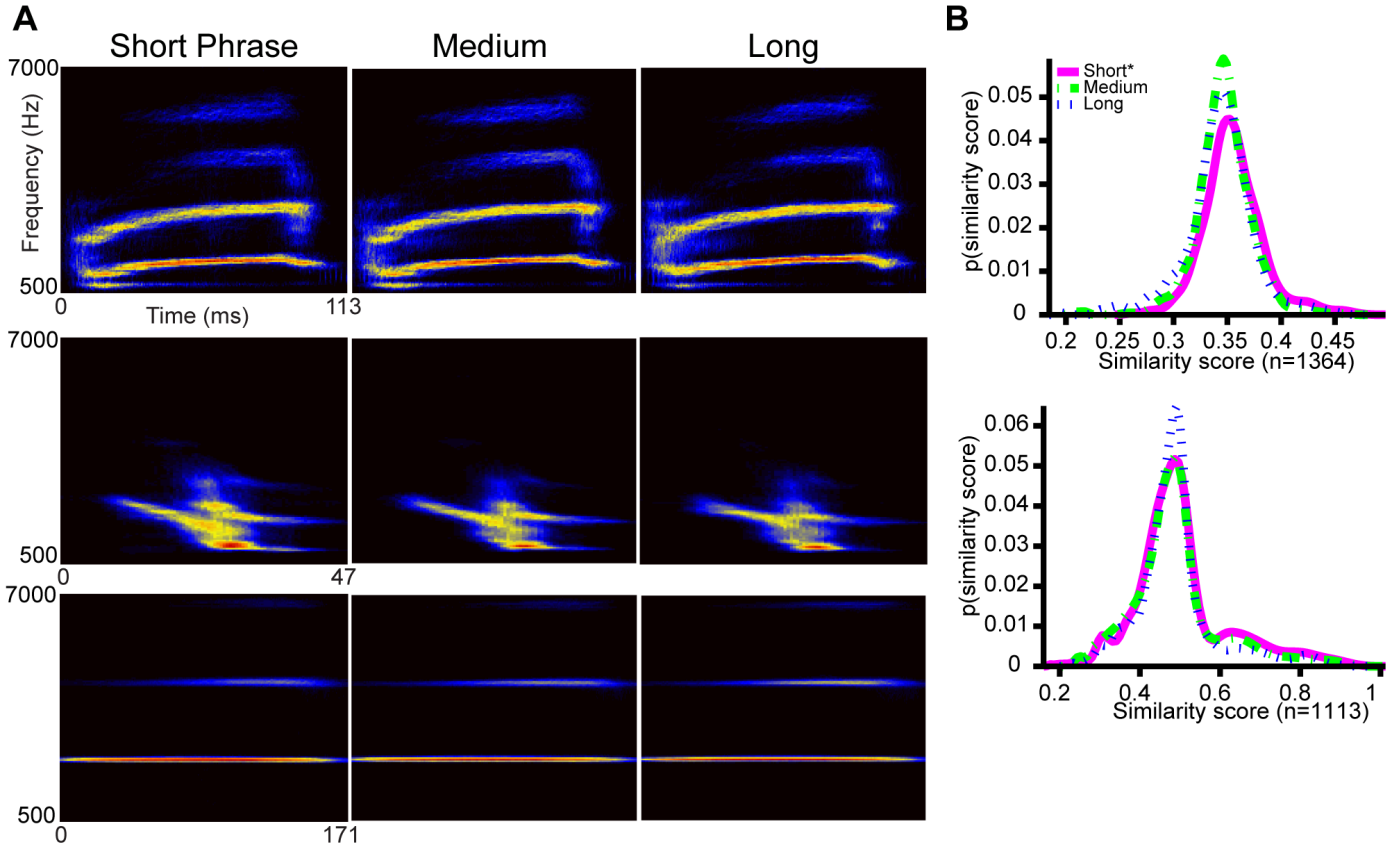
Renditions of the same syllable in short, medium and long phrases are highly similar. **A,** Spectral density images were computed for matching syllables in phrases of different relative durations. The images were taken from 2 different birds. **B,** Similarity scores reflect the acoustic similarity between individual sounds and the spectral density image for a group of sounds. Here, we computed the similarity scores of the same syllable extracted from short, medium and long phrases with respect to the spectral density image for syllables extracted from short phrases. These distributions show that phrase duration does not affect the acoustic form. *Top:* similarity scores for the syllable type from the middle row in **Fig. 3a**. *Bottom:* similarity scores for the syllable type from the third row in **Fig. 3a**. Summary statistics for these distributions are given in **Table S2**.

Renditions of what appeared to be the same syllable in short and long phrases might show subtle distinctions acoustically. If they are distinct, then the apparent long-range correlations could be more simply described by nearest neighbor rules in the syllable sub-types [16]. Here we sought to provide a methodology for direct visual representation of syllable variability. To proceed, we first isolated all renditions of a chosen syllable based on an automated template matching procedure (see **Materials and Methods**). We confirmed by visual inspection of sonograms that all examples of the chosen syllable were extracted, with no errors. We then separated the syllables into three groups depending on whether they came from short, medium or long phrases. (Phrase durations were discretized into three bins of uniform time-span, an arbitrary choice.) We then generated spectral density images (see **Materials and Methods**) for each group. The spectral density image provides a quantitative representation of syllable form and its variability across multiple renditions. In the spectral density image, color scale indicates the probability of finding a time-frequency contour [25] at a given point in the time-frequency plane. For a few syllables, visual inspection of the spectral density image revealed no variation for syllables drawn from different phrase duration bins (**Fig. 3a**).

This qualitative observation can be quantified using a similarity score based on the spectral density images (see **Materials and Methods**). Specifically, we computed the all-to-all overlap of binary contour images by computing the inner product between all pairs. Then, the all-to-all scores were sorted by their corresponding phrase groups and the distributions were compared using a Kolmogorov-Smirnov (KS) test and a d′ measure (see **Materials and Methods**). By the KS test, the distributions were distinct (p<.01), however the scale of the acoustic differences was very small by our measure. In all cases d′<0.2 (see **Materials and Methods**), indicating that the average differences between syllable shapes in different groups were smaller than the variations within a given group (**Fig. 3b**, and **Table S2**). Corroborating measurements were found using scores computed from acoustic features defined in the Sound Analysis Pro for MATLAB package [26]. For these scores, d′<0.1 (see **Text S1**).

In this analysis, we chose the most stable syllables. In other syllable types (particularly the fastest syllables), a systematic shift in acoustic form may occur over the course of a phrase. Also, for many phrase types in canaries, the first syllable of a new phrase type shows a structure that matches neither the preceding nor the succeeding phrase. If a switch in syllable forms is made in the central motor control loops, the syringeal or respiratory pattern may require a finite time to reconfigure. Ongoing phonation during this period of reconfiguration may produce syllable forms that differ from the steady state syllable forms [27]. **Fig. S10** reveals that these specific context dependent effects can be acoustically significant. Excluding the special case of the first transitional syllables in a new phrase, in the syllables analyzed here, changes in form in different contexts were too small to allow a single instance of a syllable to be reliably assigned to short, medium, or long phrases using scores based on either SAP features or spectral density images.

### Second order Markov structure of phrases

**Figure 4:**
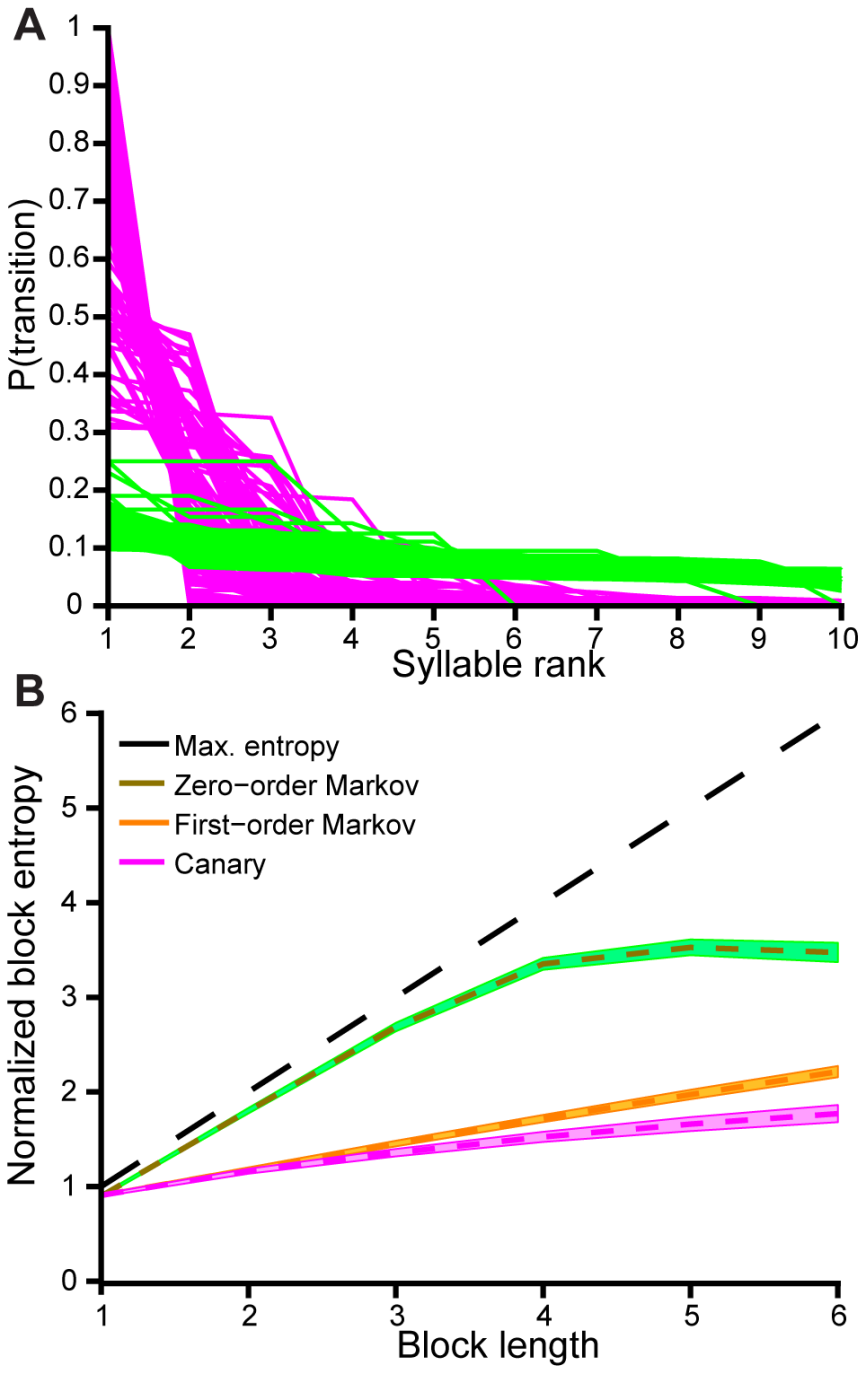
Canary phrase sequences are more ordered than a first-order Markov process. **A,** For each phrase type the magnitudes of transition probabilities to the next phrase are shown sorted by probability. The rapid decay of the traces indicates that most syllables are followed by just a few high probability transitions. Canaries (magenta) do not simply emit phrases at random, as in the zero-order Markov case (green). **B,** Block entropy is the entropy computed for sequences of *n* phrases, i.e. the block size (see **Materials and Methods**). The block entropy was computed for the actual phrase labels (canary), a randomization that only preserved phrase occurrence probabilities (zero-order Markov), and a randomization that only preserved nearest-neighbor transition probabilities (first-order Markov). Block entropy grows slowly with block size (n) for canary song, relative to the random models. This slower entropy growth hints at the existence of structure beyond a first-order Markov chain in phrase types.

Canary song is organized around a mesoscopic structure, the phrase. Is the larger sequence of song explained by a first-order Markov process in phrases, or is phrase sequencing more complex than a first-order Markov process? To examine this possibility, we first observed that the succession of phrases is quite constrained—each phrase is typically followed by just a few downstream possibilities (**Fig. 4a**). The top three or four transitions account for most of the variations that follow a given phrase. We next examined the entropy of phrase sequences of various lengths, and compared this entropy with random sequences that preserve only first-order transition statistics. We found that the entropy of phrase sequences is almost as high as a first-order model would imply (**Fig. 4b**). However, the match is not perfect, and for sequences 4–6 phrases long, it is clear that the set of song sequences is smaller than the set of possible sequences in a first-order random model. Song is thus more ordered than a first-order Markov process acting on phrase types.

To examine further the constraints placed on canary phrase sequences, we first tested all phrase types for statistically significant second-order structure. Specifically, for a sequence of three phrases XYZ, we asked whether the phrase type X impacted the phrase type probabilities for Z, for a given phrase type Y. The test reveals that this mutual dependence exists for 70% of all examined phrase types (p<.001, Fisher-Freeman-Halton test, see **Fig. S1**). Here too, a high-resolution analysis of selected examples using spectral density images confirmed that the acoustic form of the syllable in position Y can remain relatively constant even when flanked by diverse phrase types in position X or Z (**Fig. S5a**). As for the earlier analysis of syllable forms in phrases of different lengths, this observation was quantified both with spectral density similarity scores (d′<0.35) and through the use of SAP scores (d′<0.1), indicating that variations of syllable form within each group are larger than the separation between syllable forms in different syntactic contexts. (As before, there are detectable differences between the distributions, p<.01 two-sample Kolmogorov-Smirnov test).

It is not known whether peripheral motor variables such as air pressure or muscular tone change over the course of a long canary song. Time-dependent changes in the periphery could impact the acoustic details of song [27]–[29]. We examined how syllable form changed when syllables occurred early or late in song, for a fixed phrase context defined by the immediate preceding syllable. Pairwise similarity analysis was performed for all syllables examined in the phrase-context analysis described in the preceding paragraph. Grouping syllables into renditions that occur before or after the median song duration for a given bird, detectable differences in acoustic form could be found between groups for all syllable types analyzed (p<.01 two-sample Kolmogorov-Smirnov test, n = 3 syllable types). The d′ value of the group differences is comparable to the changes reported in the previous paragraph for phrase context. For song position effects, spectral density based similarity scores reveal d′<0.2 and for SAP similarity scores d′<0.1. To summarize the analysis of syllable stability: a memory for past phrase choices impacts future phrase choices or phrase durations, and this memory may have a very limited impact on the acoustic form of some syllables (see **Fig. S5b** and **Table S3**). It is possible that the minor acoustic changes in syllables can be largely explained by small-scale drift in peripheral control variables.

### The long-range order of canary song sequences

The previous analysis indicated that second-order correlations introduce a statistically detectable shift in phrase transition probabilities for most phrase types, but these second-order effects could be weak. Still, weak higher-order correlations could in principle explain the gap between a first-order random model and canary song. However, the next stage of analysis revealed that while higher-order correlations are weak for many phrase types, for some phrase types, strong long-range rules apply to the delivery of song.

**Figure 5:**
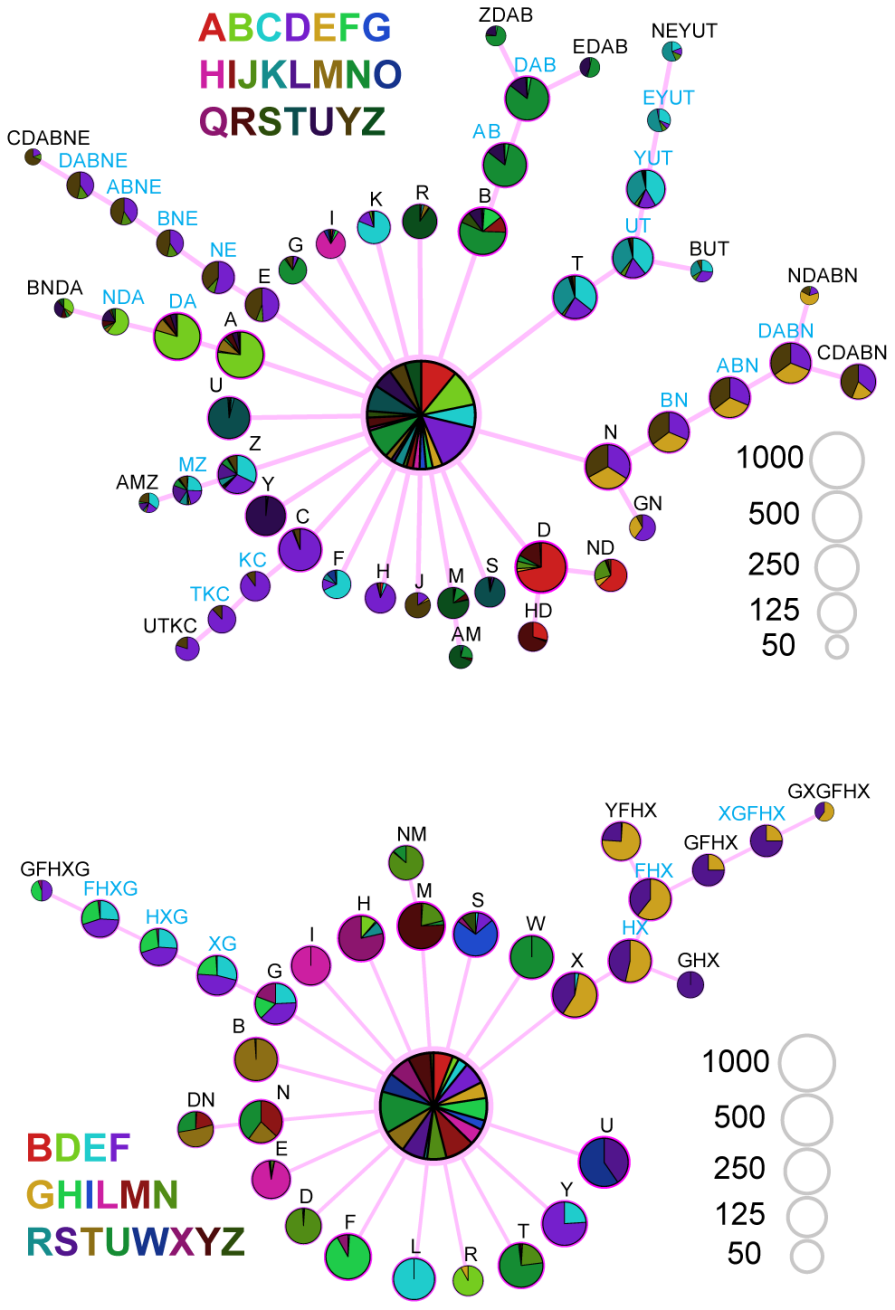
Prediction suffix trees (PSTs) reveal long-range structure in canary song. Letters are arbitrary labels for phrase types. PSTs were generated for all 6 birds (the other four are shown in **Fig. S8**). The length of the branch terminating in a given phrase type indicates the extent to which syllable history impacts transition probabilities. A branch 5 nodes long indicates that one must look 5 phrases back in the song to accurately predict the transition probabilities from the terminal node at the center. Each node is shown as a pie chart representing the outgoing transition probabilities from that sequence of phrases (for each bird, syllables are assigned arbitrary colors). The nodes are scaled according to their frequency. Nodes that can be grouped together (chunked) without significantly reducing the power of the model are labeled with blue text.

To examine how long-range correlations varied by phrase type, we constructed a prediction suffix tree (PST) [30] to represent each bird's song. A PST provides a visual representation of how past information in a sequence impacts transition probabilities. Formally, the tree is built from a collection of Markov chains, one for each phrase type. Each chain is initialized as a zero-order Markov chain, and the order is increased only if the information gained by looking further back in time justifies the added complexity (see **Materials and Methods**). In Fig. 5, the PST is displayed radially, with each syllable arranged around the inner circle. For a given syllable, the number of nodes between the tree trunk and the outer branches indicates the order of the Markov chain for that syllable. For a syllable impacted by high-order correlations, that syllable on the trunk of the PST tree will be connected to long, multi-branched limbs. Similar methods were recently used in the analysis of Bengalese finch syntax [15].

**Figure 6:**
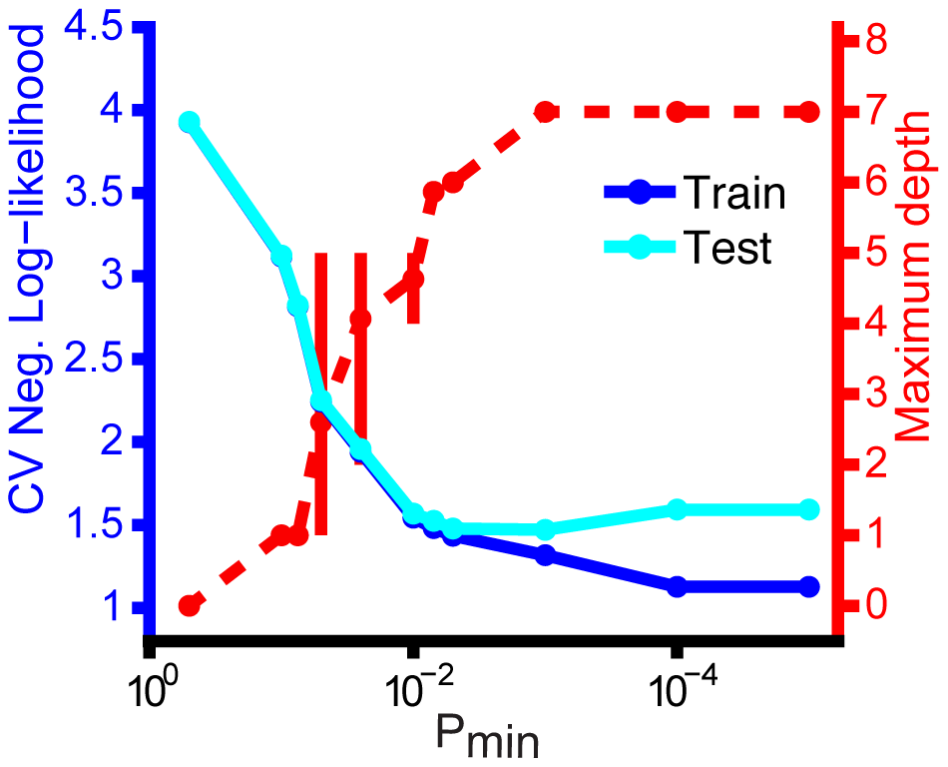
Cross-validation confirms that prediction suffix trees (PSTs) are not overfitting the data. PSTs were fit to 90% of the data and tested on the held-out 10% in a 10-fold cross-validation procedure for different values of 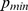, which sets the minimum frequency a sequence has to occur to be considered. If 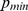 is too small, overfitting is guaranteed, and suffix trees will appear artificially deep. Shown is the performance of the PSTs for a single bird (PST is given in the bottom of **Fig. 5**). Using average negative log-likelihood to measure performance, the test performance peaks at 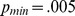. Below this value the test performance begins to degrade and sharply diverges from training performance, a clear sign of overfitting.

**Figure 7:**
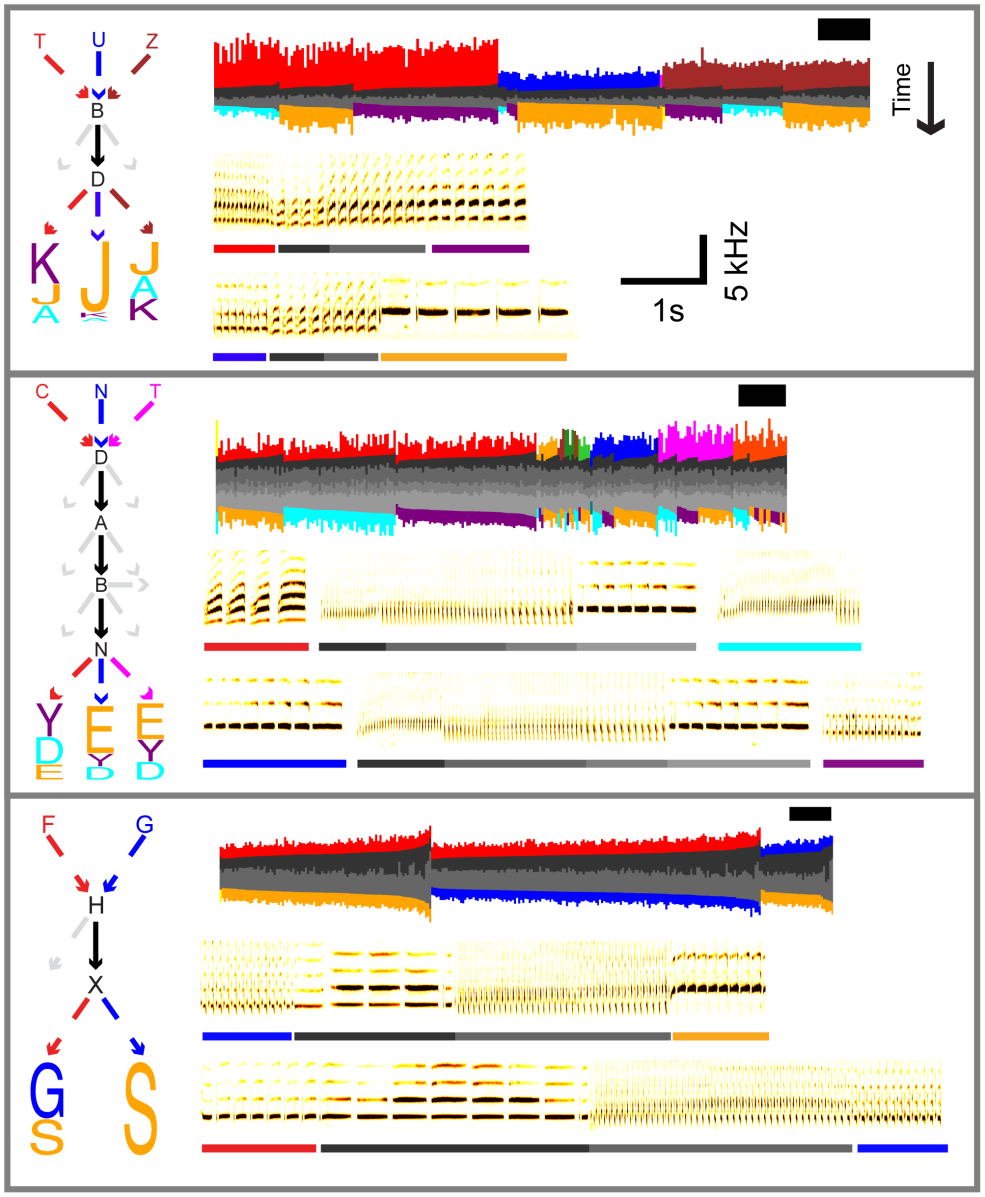
Decisions at branch points in song can impact syllable transition probabilities 5 phrases later. Each panel corresponds to a different bird. For a given panel, the colors in each part of the panel signify the same syllables. *Left:* letters (arbitrarily chosen for each bird) indicate a single phrase type, and arrows indicate phrase transitions. Entry and exit paths are color-coded and the exit paths are sorted and scaled by their transition probability. For instance, on the far left, if the bird sings T→B→D, then K is most likely to follow, whereas a J would most likely follow if he sang U→B→D. The light dotted arrows indicate other possible paths of exiting a block with p>.05. For these other paths, the destination syllable is not shown. Actual transition probabilities with 95% bootstrap confidence intervals, and the number of transitions are given in **Table S4**. *Top right*: song barcodes illustrate this effect for all occurrences of the phrase-block analyzed on the left. A square flanks each barcode to indicate the scale, with the height corresponding to 2 seconds and the width to 20 trials. Barcodes of the full song sequences are given in **Fig. S6.** *Bottom right:* example sonograms provide examples of how the entry path to a block of phrases changes the exit path.

As a control, we used a 10-fold cross-validation procedure. Prediction suffix trees were computed for the training data, and then the average negative log-likelihood of the test data was computed for each tree (**Fig. 6**). The PST that leads to the minimum in average negative log-likelihood on the test set is considered the best fit. The depth of the best fit ranged from 4 to 7 phrases in the six birds examined here (**Fig. S7**). This corresponds to a propagation of song information over a time-scale of approximately 5 to 10 seconds. Many syllables in this analysis showed no significant structure beyond first-order; just a few syllables are governed by long-range rules. (The prevalence of second-order structure revealed in **Fig. S1** suggests that the PSTs provided a conservative estimate of statistical depth for many syllables.) The structure of example songs with long time-scale correlations is illustrated in **Fig. 7** where the syllable identity of the phrase at the top of a chain impacts transition probabilities many phrases later. (**Fig. S6** contains similar song barcodes showing the full song context for the examples in **Fig. 7** **.**) In the examples given in **Fig. 7**, the history of previous syllable selections can impact future syllable transitions over 4–5 intervening phrases, spanning a time-scale of up to ten seconds.

### Reducing PST Markov order through phrase chunking

The apparent statistical depth of the phrase structure could be reduced when sequences of phrases occur as a unit [15], [16]. In sequence DABN for example, the choice of syllable D impacts choices after N, implying a fourth-order correlation. However, the PST transition probabilities for nodes A, B and N all have equivalent or near-equivalent values. The state space can be reduced to D (ABN)—a second-order model in phrase sequence “chunks.” In the process of constructing a PST, the nodes that can be collapsed into a single chunk without changing the predictive power of the model are “internal nodes.” These nodes do not impact the transition probability at the end of a chain, but just provide a connection from the leaves of the tree to the trunk. In **Fig. 5** and **Fig. S8** internal nodes are labeled with blue text labels. (Here internal nodes are defined as those nodes that would not have been added to the PST on their own strength, but are simply added to show the connections from the outer branches to the core of the graph. As such, the definition of internal node depends on the parameters used in the PST fit.) After collapsing internal nodes in these figures, the maximum depth of the suffix trees reduce from a range of 4–7 to a range of 2–3 in the 6 birds analyzed here, whereas the log-likelihood of the model changed, on average, by less than 1 percent, indicating that sequence chunks could be regarded as monolithic states without impacting the quality of the model.

### First order models

**Figure 8:**
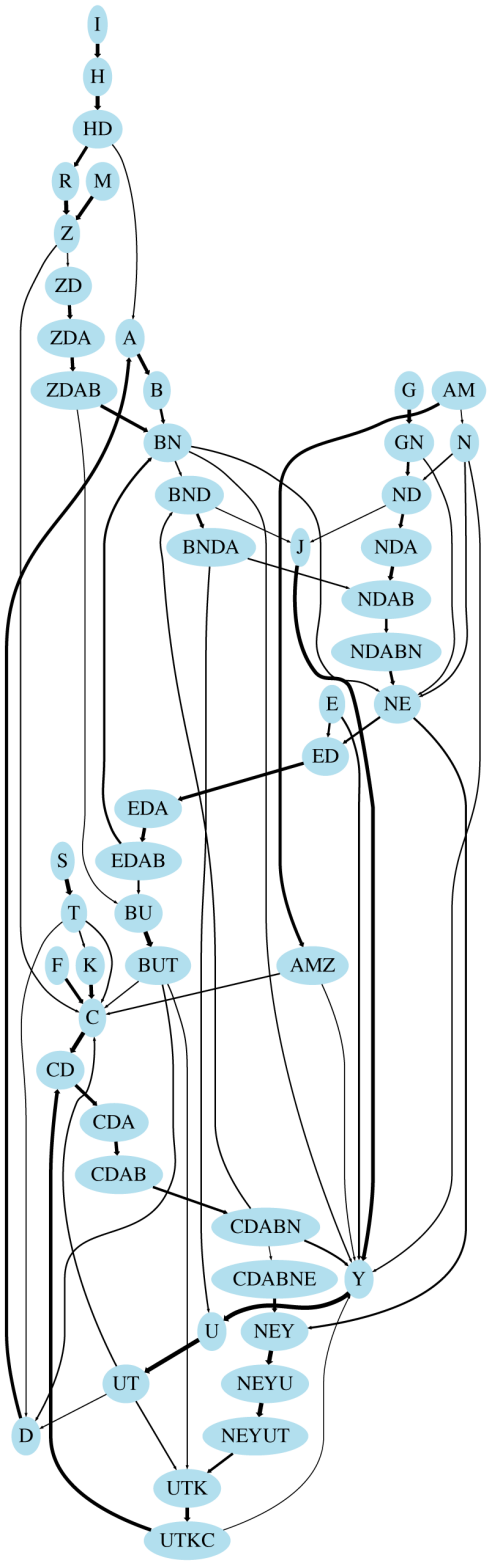
Probabilistic finite automata (PFA) computed from prediction suffix trees (PSTs) reveal the complexity of canary song. The PFA shown here corresponds to the PST from the top of **Fig. 5**. Edges represent transitions between states, and the width indicates the probability of a given transition. Transitions from one state to another always result in the production of a new phrase type. For instance, the edge connecting ZDAB to BN represents the probability of singing N after ZDAB, which leads to the state BN. All edges with transition probabilities below .2 were removed for visualization (see **Fig. S11** for a more complete PFA).

The PST provides a particularly compact representation of long-range dependencies in song. The compactness of the PST representation is emphasized by comparison of the PST graph with its corresponding probabilistic finite automaton (PFA) [30]–a first-order transition model that can be more easily related to first-order dynamical models of neural activity. **Fig. 8** illustrates the graph structure of the PFA for one bird, whose PST is given in the top of **Fig. 5**. To render the PFA visually interpretable, 363 edges were deleted from this figure that occur with less than 20 percent probability (more complete PFAs are shown in **Fig. S11**). We emphasize that in spite of the complexity of **Fig. 8** and **Fig. S11**, the PFA is only a statistical model for *phrase transitions*. The model does not account for phrase *durations*, or the fact that phrase durations depend on the recent history of the song–a point documented earlier–these features should be addressed in a more complete statistical model.

### Tests for simple patterns

We next examined whether the long-range correlations followed a simple adaptation rule. Long-range correlations could appear if the probability of a given phrase transition decays with the frequency of its use. That is, as each phrase transition occurs, its subsequent probability of occurrence is decreased. In the cases examined in **Fig. S9**, no simple rule was observed—the most frequent syllable transition from a given phrase can increase or decrease in likelihood as a function of the number of times the phrase is produced in a given song. Over the transitions that we could analyze for this property, 33% strictly increased while 50% decreased, and 13% both increased and decreased over the course of a song (17/52, 26/52 and 7/52, respectively).

We then checked to see if the statistical depth of canary song could be explained by limitations on song duration. The concern here is that some branches in the path of song might lead to unusually long songs that could be prohibited for physiological reasons. We analyzed all examples of context-dependencies and found no evidence for this effect (see **Text S1**). This search for simple rules explaining the apparent depth of canary song was not exhaustive, and a more extensive analysis involving additional samples from other canaries is needed.

## Discussion

Canary song is built from elementary units, the syllables, repeated in groups to form a mesoscopic structure, the phrase. Phrases are flexibly sequenced to form songs. Correlations among phrase choices can extend over time-scales of 7–10 seconds. Over this time-scale, 4–5 phrases may be produced consisting of typically dozens of syllables. This observation significantly extends the time-scales that must be considered in dynamical models for song generation. We first discuss the time-scale of a single phrase. Dynamical theories for the central control of song are, in various forms, attractor models [31]–[36]. If each phrase type is a separate attractor (or closed-neural chain) in canaries, then the phrase transition could be produced by a “kick” that recurs every second or so, inducing a hop from one attractor to another. Statistically, the phrase durations of canary song could also be described by a POMMA model [16] if each syllable has a self-return probability that decreases with each repeat, as long as the adaptation rate scales inversely with syllable duration. More simply, phrase time-scales could be introduced into first order models like POMMA by introducing an adaptation that changes as a function of time rather than syllable repeats. Experiments are needed to determine whether canary phrase time-scales are defined by a fine-tuning of syllabic adaptation rates or a separate phrase transition process with its own intrinsic time-scale. Whatever the mechanism of defining phrase durations, it apparently does not need to be informed by any auditory experience with natural canary song, since birds reared in acoustic isolation also develop canary-typical phrase structure [22].

The observed structure of canary song significantly extends the time-scale of long-range correlations documented in bird song. For example, the sequence CDABNE from the top of **Fig. 5** has an average duration of eight seconds and the sequence YFHX from the bottom of **Fig. 5** has an average duration of ten seconds. Long-range rules in canary song can be compactly described, by 4^th^–7th order Markov processes acting on phrases, or a 2^nd^–3^rd^ order Markov process acting on larger units that include blocks of multiple phrases.

A suffix tree of depth 7 for 30 phrase types could in principle have 30∧7 nodes, and even a third order process in 30 phrase types could have 30∧3 nodes. In contrast to this large state space, the PSTs included in **Fig. 5** are quite sparse. The first-order generative models for the canary songs represented by the PFA diagrams contain only 47–56 states representing syllables or syllable strings. This is not greatly larger than the number of observable syllables (17–26). For canary song, the PST provides a particularly compact representation of syntax dependencies. The compactness of this representation is clear in comparisons between the PST tree and its corresponding first-order models (**Fig. S11**). The speed and convergence properties of the PST algorithm make it possible to quickly cross-validate the structure of the PST over large ensembles of trials, defining the point at which over-fitting occurs. Taken together, these properties suggest that the PST analysis will be generally useful for characterizing the structure of birdsong syntax.

Statistically complex phenomena that are best described by higher-order Markov processes such as the PST can be generated by simple physical processes. For example, if circulating neuromodulators in song nuclei depend on the syllables that are sung, and if syllable transitions themselves depend on the hypothetical neuromodulator, then stochastic variations in the beginning of a song could impact future transition probabilities, generating apparent “long-range rules”. In this scenario, additional information is needed to explain why some syllables show strong long-range rules, and others behave in a simple first-order manner.

On a fine-grained scale, neural dynamics should be captured by a first-order statistical process. In statistical terms, long-range correlations in syntax imply that multiple “hidden states” can give rise to the observable syllable. This duplication of states statistically does not imply that the motor program for a syllable is duplicated–the smallest change in a syllable program satisfies the duplication of “hidden states.” Recent studies in Bengalese finches have observed that the stereotyped neural program for a syllable depends on its context–in particular, changes were observed in Basal Ganglia projecting neurons in nucleus HVC (used as a proper name) [37], and subtle acoustic changes in syllable form were observed for syllable in different contexts [16]. Whatever the mechanism of the long-range rules in canaries, the neural variables that carry the memory for past song choices can exert a powerful effect on transition statistics without significantly altering the acoustic form of syllables. This observation is supported through the high-resolution “spectral density” images introduced here to characterize syllable variability.

Canary phrase structure and canary syllable form appear to be encoded by separable processes. This distinction is supported by the observation that phrase time-scales are not simply predicted by syllable time-scales. Another line of evidence arises in studies of song learning in juvenile canaries. As juveniles, canaries can learn to imitate artificial songs that lack normal phrase structure [22]. Rising testosterone levels that occur with the onset of the breeding season cause a rearrangement of song–the imitated syllables are reorganized into phrased repetitions. In this artificial tutoring paradigm, what is most dramatically reprogrammed in the transition to adulthood is not syllable acoustic structure, but the sequential organization of the syllables.

Many questions remain about the neural basis of phrase structure in canary song, but we may also wonder about the relevance of the long-range rules for the natural behavior of the species. Is the statistical depth of song an epiphenomenon of little ethological relevance, or do canaries show preferences for songs with long-range order? Can a canary fine-tune the long-range rules to match a tutor song, or can a bird be trained to alter rule-sets in different behavioral contexts? These questions are addressable since canaries readily imitate artificial songs designed to pose specific questions about their vocal learning processes [22].

Lashley emphasized that the control of serial order in behavior is one of the most important and least understood aspects of neuroscience over 60 years ago [38]. Songbirds have provided an opportunity for examining sensory-motor learning of stereotyped neural sequences; dynamical models for song sequence generation have generally focused on stringing together bursts of neural activity in a long chain of elementary states [34], [39]. This representation is remarkably similar to observed neural dynamics in zebra finches and Bengalese finches, and can be related to simple first-order statistical models for song production [16], [17]. However, the long-range rules that govern canary song extend to time-scales of 10 seconds, and persist while a bird vocalizes five or six intervening phrases, consisting of dozens of syllables each. How is information in the song circuit transferred over these time-scales? Answers to this question may provide general principles of how complex behaviors with long-range correlations are assembled from simple modules.

## Materials and Methods

### Ethics statement

The care of animals in this study was carried out in accordance with Boston University IACUC protocol number 09-007.

### Song recording

Canaries (Belgian Waterslager strain) used in this study were a gift from Fernando Nottebohm. Birds were isolated at least two weeks before recording in soundproof boxes and kept on a light-dark cycle matched to the external annual light cycle in Boston (Boston University IACUC protocol number 09-007). All birds were at least one year old before isolation. Song was recorded between the months of March and April.

### Song labeling and software

Spectrograms of the song were calculated in MATLAB (Mathworks, Natick, MA), and the beginning, end, and syllable identity of each phrase was marked on the image by visual inspection. For all data described here, two independent observers annotated the songs. Observer 1 and observer 2 annotated 33,469 and 36,447 phrases, respectively, between 6 birds (see **Table S1** for the number of phrases analyzed for individual birds). The annotated sonograms were then scanned and converted into strings, and statistical analysis of the strings was performed using custom MATLAB scripts. Zero to two syllables were excluded per bird because the syllable form consisted of subtypes that could not be labeled consistently.

### Mutual dependence analysis

For each bird (6 total), we first examined the mutual dependence between a given phrase's duration and the path into or out of the phrase. That is, in a given phrase sequence XYZ, we examined the mutual dependence between the length of phrase Y and identity of the syllable type in phrase X or the identity of the syllable type in phrase Z, which we call MD(dur,path_in_) and MD(dur,path_out_), respectively. We discretized phrase durations by terciles and then used a variation of the Fisher test for 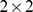 contingency tables suitable for arbitrarily large, sparse 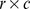 contingency tables referred to as the Fisher-Freeman-Halton test [40]. Significance values for the test were then computed using a standard Monte Carlo procedure where the contingency tables were randomized while preserving the marginal values (i.e. the row and column totals), and to derive a p-value we calculated 1,000,000 randomizations for each test [41]. Using the same method, we also examined the mutual dependence between the syllable identity of phrase X and the syllable identity of phrase Z, for each Y, which we label MD(path_in_,path_out_).

### Automated syllable clustering and alignment

Custom MATLAB (Mathworks, Natick, MA) scripts were used for automated syllable clustering. After choosing a template, spectral features from the template and the rest of the data were computed using a sparse time-frequency representation [42] (see **Text S1**). As a final step, candidate sounds were plotted in two dimensions and a decision boundary was drawn by the user.

### Spectral density images and similarity scores

For a collection of syllables, we first generate a sparse time-frequency representation of each syllable using auditory contours [25]. Auditory contours provide a high-resolution binary image consisting of sparse, continuous lines that follow the features of the sound with high precision. Summing over contours for all renditions of a syllable produces a two-dimensional probability density in time and frequency, which we call a spectral-density image, 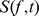. Specifically, a single contour image calculated at a given resolution is a sparse, binary time-frequency image or spectrogram 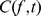, which we compute for each syllable 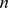, denoted 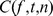. The spectral density image is then defined as 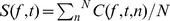, where N is the number of binary images. By definition, 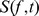 is the probability of finding a contour in pixel 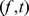 in a single sample, and in the images shown here, the value 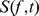 is represented by color scale. These images provide a direct representation of the variability of syllable form for every point in time and frequency.

Since the starting matrices 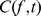 are sparse, and high precision, the spread of the acoustic energy revealed in 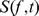 may be narrower than the resolution of standard sonograms, for sparse stereotyped song elements. When interpreting these images, it must be understood that variations in frequency or timing of auditory contours both lead to a spread in spectral density; separating these sources of variability is not generally possible without additional analysis such as time-warping [22].

We define a simple similarity measure between two syllable contour images 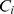 and 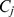 to be 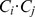; With this definition, the average similarity between two groups **A** and **B** is just 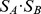 , the inner product of their spectral density images. The average similarity between a one specific syllable 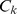 and an ensemble of syllables **B** is 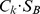. To quantify the separability of two distributions we use the d′ measure, 
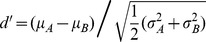
, or the difference in mean similarity scores in units of pooled standard deviation.

### Prediction suffix trees

To test for the existence of structure beyond second-order (as in the MD(path_in_,path_out_) test), we constructed prediction suffix trees (PSTs) for the sequence of canary phrase types using a previously published algorithm [30], [43]. Further details can be found in **Text S1**.

### Block entropy

To compute block entropy we used standard methods based on Shannon entropy [44]. The maximum entropy line in **Fig. 4** assumes symbols are emitted with uniform probability.

## Supporting Information

Audio S1 **Sample series of song bouts from a single canary.**
(MP3)

Audio S2 **Sample series of song bouts from a single canary, separate from the bird used for Audio S1.**
(MP3)

Figure S1
**Pie charts of p-values for association tests.** Charts computed using data from two observers are shown for six birds (each column is a different bird). The graphical conventions of the pie charts follow **Fig. 2b** from the main text. Yellow indicates the proportion of syllables with highly significant interactions at the level 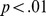, orange 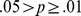 and blue 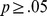.(TIFF)

Figure S2 **Scatter plots of test statistic values computed for the MD(path_in_,path_out_) test show high inter-observer agreement.** The x and y coordinates of each point are the Fisher-Freeman-Halton test statistic values computed for the same phrase type from each of the two observers for the MD(path_in_,path_out_) test. Linear regression lines are given in red and the unity line in blue, along with the r and p values under the abscissa. Each point in the graph is one phrase type for a specific bird.
(TIFF)

Figure S3 **Scatter plots of test statistic values computed for the MD(dur,path_out_) test show high inter-observer agreement.** The x and y coordinates of each point are the Fisher-Freeman-Halton test statistic values computed for the same phrase type from each of the two observers for the MD(dur,path_out_) test. Linear regression lines are given in red and the unity line in blue, along with the r and p values under the abscissa. Each point in the graph is one phrase type for a specific bird.
(TIFF)

Figure S4 **Scatter plots of test statistic values computed for the MD(path_in_,dur) test show high inter-observer agreement.** The x and y coordinates of each point are the Fisher-Freeman-Halton test statistic values computed for the same phrase type from each of the two observers for the MD(path_in_,dur) test. Linear regression lines are given in red and the unity line in blue, along with the r and p values under the abscissa. Each point in the graph is one phrase type for a specific bird.
(TIFF)

Figure S5 **Spectral density images demonstrate that syllables occurring in different contexts are highly similar.** **A,** Spectral density images of syllables with different surrounding phrases reveal acoustic similarity. As in **Fig. 3a**, spectral density images were computed for matching syllables that occur in different contexts; that is, different surrounding phrase types of earlier or later phrases. The particular phrase shown is highlighted in bold just above the image. The images were taken from 2 different birds. As in **Fig. 3a**, each row contains the same syllable type in three context groups that are acoustically indistinguishable by our statistical test. **B,** With the spectral density image from the magenta group as a reference, syllables occurring in different sequences have overlapping similarity score distributions. The syllable types match those shown in **Fig. S5a**. Summary statistics for all syllables types in **Fig. S5a** are given in **Table S3**.
(TIFF)

Figure S6 **Full song barcodes for the examples given in** **Fig. 1** **and** **Fig. 7** **allow for direct visualization of long-range rules.** These barcodes represent the full song sequence corresponding to the examples given in **Fig. 1** and **Fig. 7** **. A,** Barcodes for the example from **Fig. 1a** centered on the first occurrence of the black phrase. **B,** Barcodes for the example in **Fig. 1b** centered on the black and gray phrases. **C,** Barcodes for the example from the middle of **Fig. 7** centered on the first occurrence of N (shown here in blue). **D,** Barcodes for the example from the bottom of **Fig. 7** centered on the first occurrence of X. All colors match the phrases used in the corresponding parts of **Fig. 1** and **Fig. 7**, all other colors are arbitrarily assigned. A square flanks each barcode to indicate the scale, with the width corresponding to 2 seconds and the height to 20 trials.
(TIFF)

Figure S7
**Using cross-validation to verify PST fits.** To select the parameters used in the PST algorithm, we used a 10-fold cross-validation procedure repeated 3 times (with different data splits). As a measure of model performance we used average negative log-likelihood. Here we show cross-validation results as a function of the PST parameter 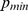. This parameter defines the minimum rate of occurrence for a sequence to be considered for incorporating into the PST. The plot shows the mean of the negative log-likelihood across cross-validation fits for the training and testing data for all 6 birds (error bars indicate the 25^th^ and 75^th^ percentiles). Also shown is the average maximum order of the PSTs for a given value of 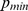. Overfitting leads to a decline in performance on the test set, or increased negative log-likelihood. In each case, the optimal performance on the test set occurs when 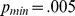 or 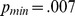. Below this point, performance on the test set degrades and sharply diverges from training set performance. We conservatively set 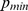 to .007 for all birds to avoid overfitting. The same procedure was used to set 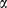 and r, which had negligible effects on model performance (data not shown).(TIFF)

Figure S8 **PSTs for the 4 birds not shown in** **Fig. 5** **.**
(TIFF)

Figure S9 **Shown are the transition probabilities from two different phrases sung by the same bird as a function of repetition number.** *Left:* as phrase X is repeated, the most probable phrase transition from X to G decays, while the transition to S increases. *Right:* the opposite effect is seen in the same bird. The most probable transition, from H to X, increases as H is repeated.
(TIFF)

Figure S10 **Some syllables have context-dependent transitional forms.** **A,** shown on the left are two example sonograms of the same phrase with different preceding phrases. In the two contexts, the first syllable of the phrase has a different transitional form (highlighted by the red and blue boxes). The image on the right is a color channel merge of two spectral density images, which were computing using the first syllable of all phrases in the two different contexts (top sonogram context is the red channel and bottom sonogram context the blue channel). **B,** the same effect is shown for a different phrase from a different bird.
(TIFF)

Figure S11 **Probabilistic finite automata (PFA) for all 6 birds analyzed.** For visualization, all edges where p<.05 have been removed, and all edges where p<.2 are shown in thin light gray lines. Each PFA is completely determined by its corresponding PST. From top to bottom, the PFAs correspond to the PST shown in: the top left of **Fig. S8**; top right of **Fig. S8**; top of **Fig. 5**; bottom left of **Fig. S8**; bottom of **Fig. 5**; bottom right of **Fig. S8**.
(TIFF)

Figure S12 **Phrase durations for all 6 birds analyzed.** Each row corresponds to a different bird (same order as **Fig. S11**), and each group of points indicates the duration of a different phrase type. The colors (arbitrarily chosen) indicate different preceding phrase types. Abrupt changes in duration distribution that co-occur with color changes reveal a context-dependent shift in phrase length.
(TIFF)

Table S1 **The total number of phrases analyzed by each observer for individual birds.** The bottom row contains the repertoire size for each bird.
(DOCX)

Table S2 Summary statistics for similarity scores for duration groups (for each syllable type, scores were computed referenced to the spectral density image from the group marked*). STD, standard deviation.
(DOCX)

Table S3 Summary statistics for similarity scores for sequence groups (for each syllable type, scores were computed referenced to the spectral density image from the group marked*). STD, standard deviation.
(DOCX)

Table S4 **Transition probabilities and number of transitions to accompany** **Fig. 7** **.** The 95% confidence intervals are given in brackets below the transition probabilities and were estimated using a bootstrap procedure with a case resampling scheme. That is, the transition probabilities were estimated after randomly resampling the data with replacement. The confidence interval is then defined as the 2.5^th^ and 97.5^th^ percentiles of the resampled distribution.
(DOCX)

Table S5 **Transition probabilities demonstrating alternative paths for prediction suffix trees (PSTs) shown in** **Fig. 5** **and Fig. S8.** The sequence DABN comes from the top PST in **Fig. 5**, HX from the bottom PST in **Fig. 5**, and ZGKLH from the bottom left PST in **Fig. S8**.
(DOCX)

Text S1 **Supplementary** **Materials and Methods** **.**
(DOCX)
