# Supplementary figures and images for: Long-range Order in Canary Song
Source: PLoS Comput Biol. 2013 May 2;9(5):e1003052. doi: 10.1371/journal.pcbi.1003052 (PMC3642045; doi:10.1371/journal.pcbi.1003052)

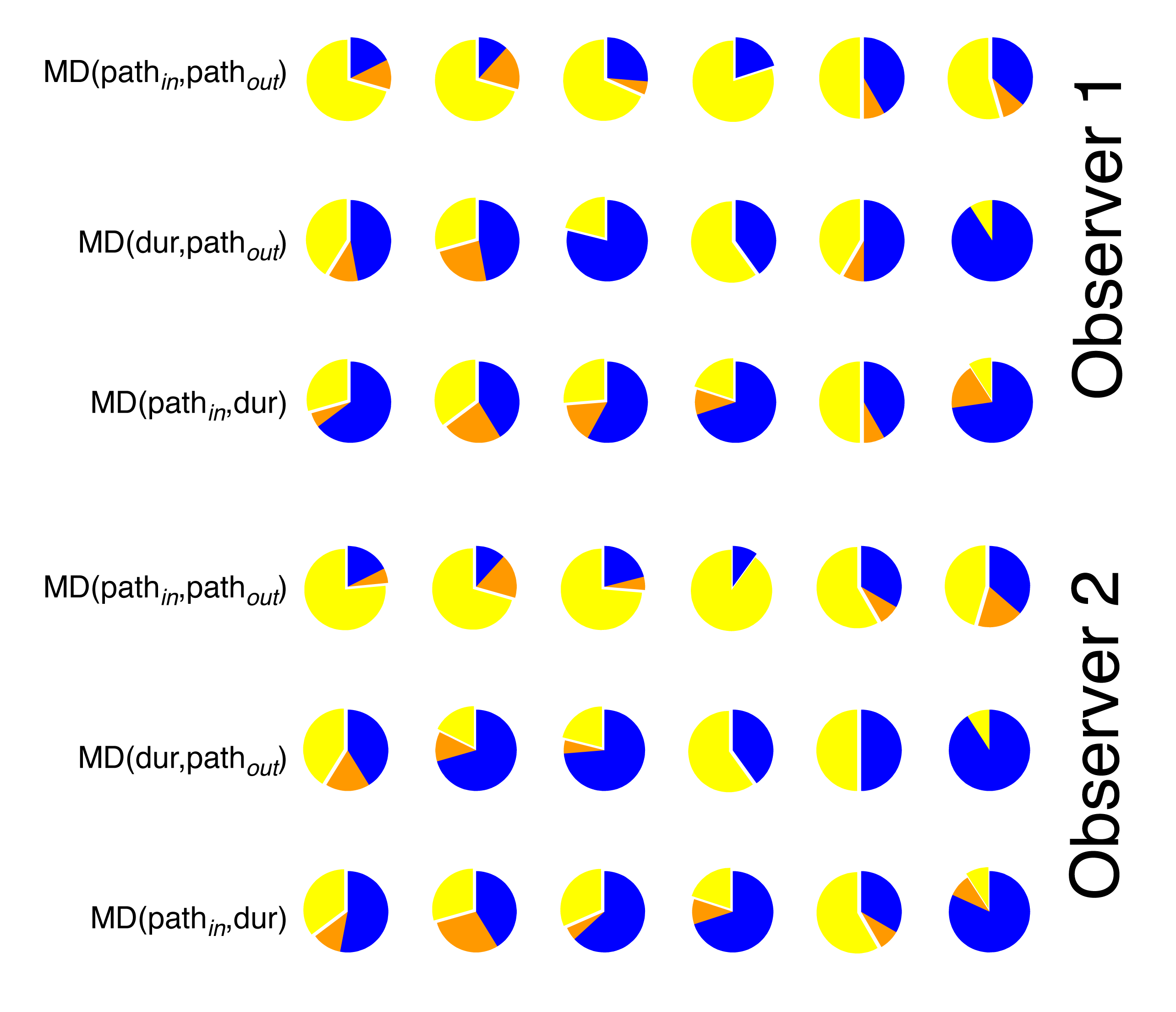

Supplement: Figure S1 — Pie charts of p-values for association tests. Charts computed using data from two observers are shown for six birds (each column is a different bird). The graphical conventions of the pie charts follow Fig. 2b from the main text. Yellow indicates the proportion of syllables with highly significant interactions at the level , orange and blue . (TIFF) [file pcbi.1003052.s003.tiff]

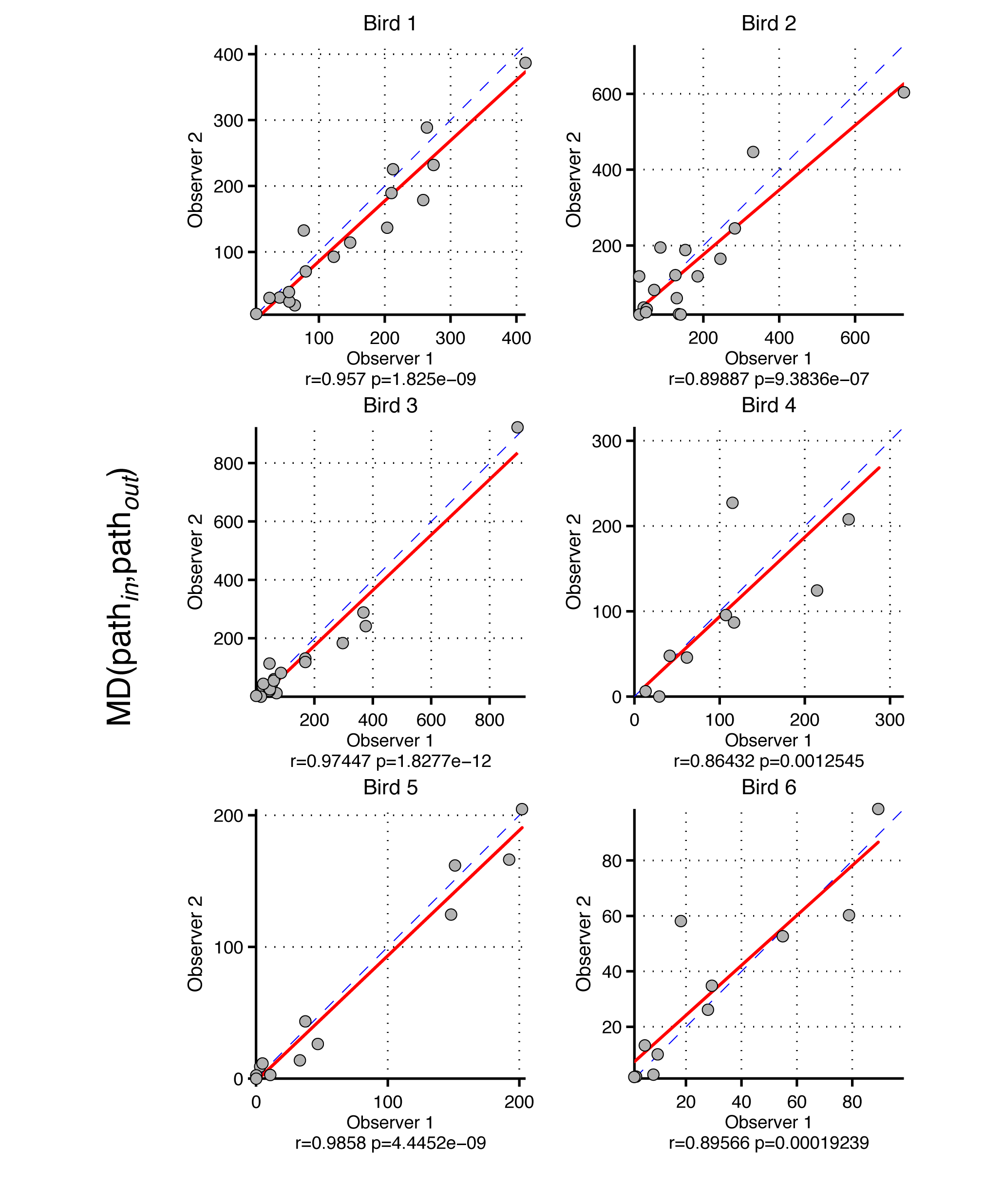

Supplement: Figure S2 — Scatter plots of test statistic values computed for the MD(pathin,pathout) test show high inter-observer agreement. The x and y coordinates of each point are the Fisher-Freeman-Halton test statistic values computed for the same phrase type from each of the two observers for the MD(pathin,pathout) test. Linear regression lines are given in red and the unity line in blue, along with the r and p values under the abscissa. Each point in the graph is one phrase type for a specific bird. (TIFF) [file pcbi.1003052.s004.tiff]

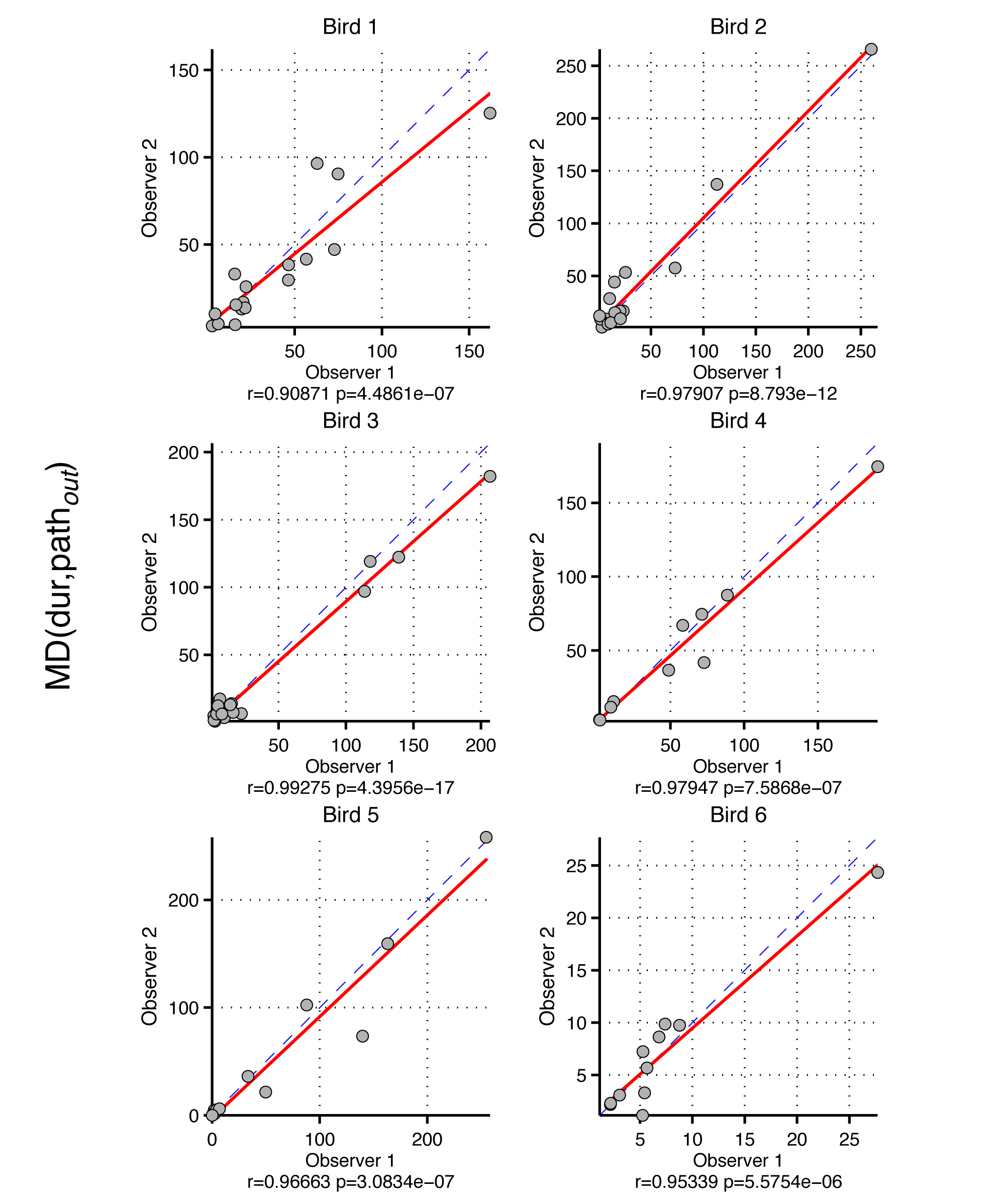

Supplement: Figure S3 — Scatter plots of test statistic values computed for the MD(dur,pathout) test show high inter-observer agreement. The x and y coordinates of each point are the Fisher-Freeman-Halton test statistic values computed for the same phrase type from each of the two observers for the MD(dur,pathout) test. Linear regression lines are given in red and the unity line in blue, along with the r and p values under the abscissa. Each point in the graph is one phrase type for a specific bird. (TIFF) [file pcbi.1003052.s005.tiff]

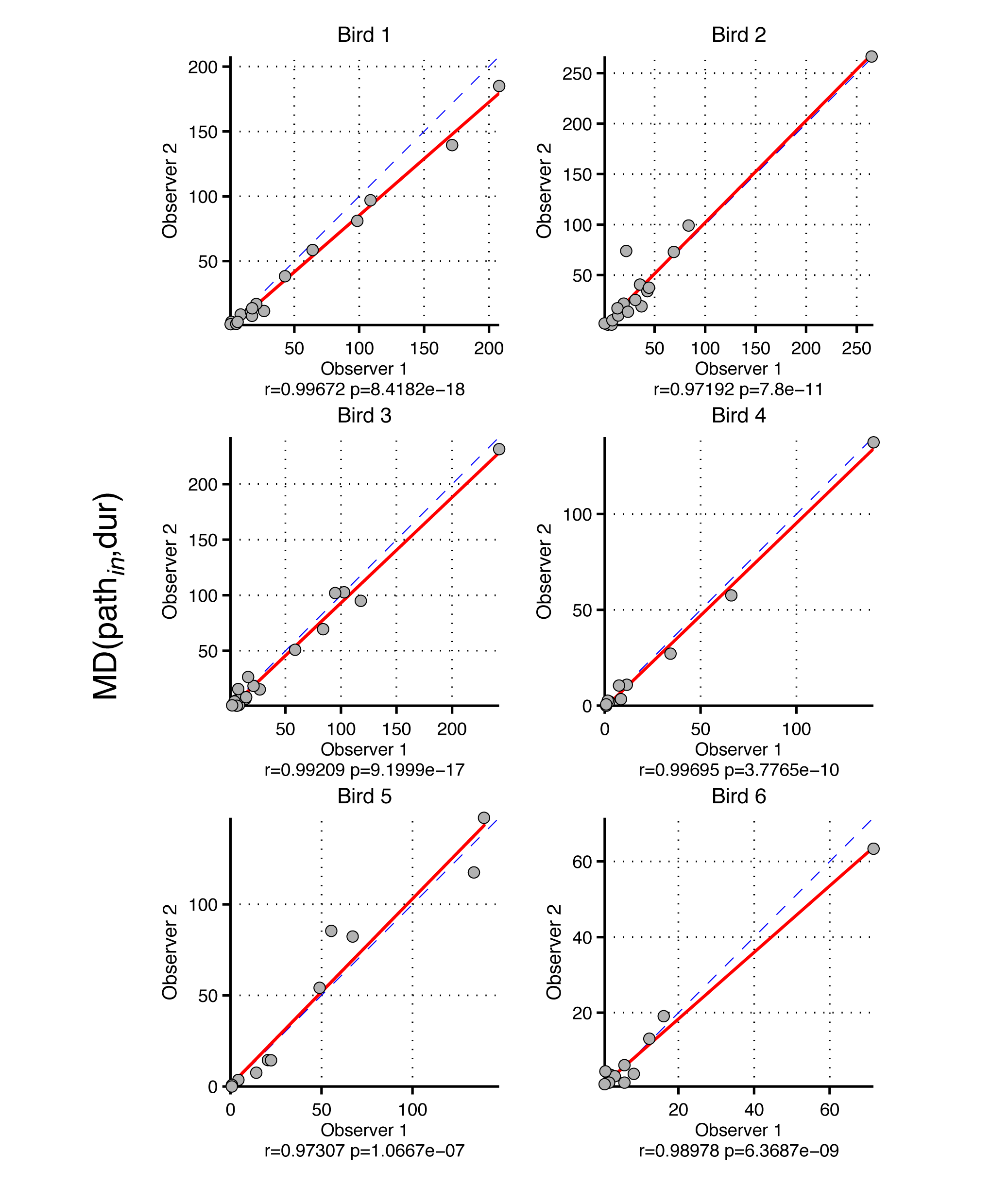

Supplement: Figure S4 — Scatter plots of test statistic values computed for the MD(pathin,dur) test show high inter-observer agreement. The x and y coordinates of each point are the Fisher-Freeman-Halton test statistic values computed for the same phrase type from each of the two observers for the MD(pathin,dur) test. Linear regression lines are given in red and the unity line in blue, along with the r and p values under the abscissa. Each point in the graph is one phrase type for a specific bird. (TIFF) [file pcbi.1003052.s006.tiff]

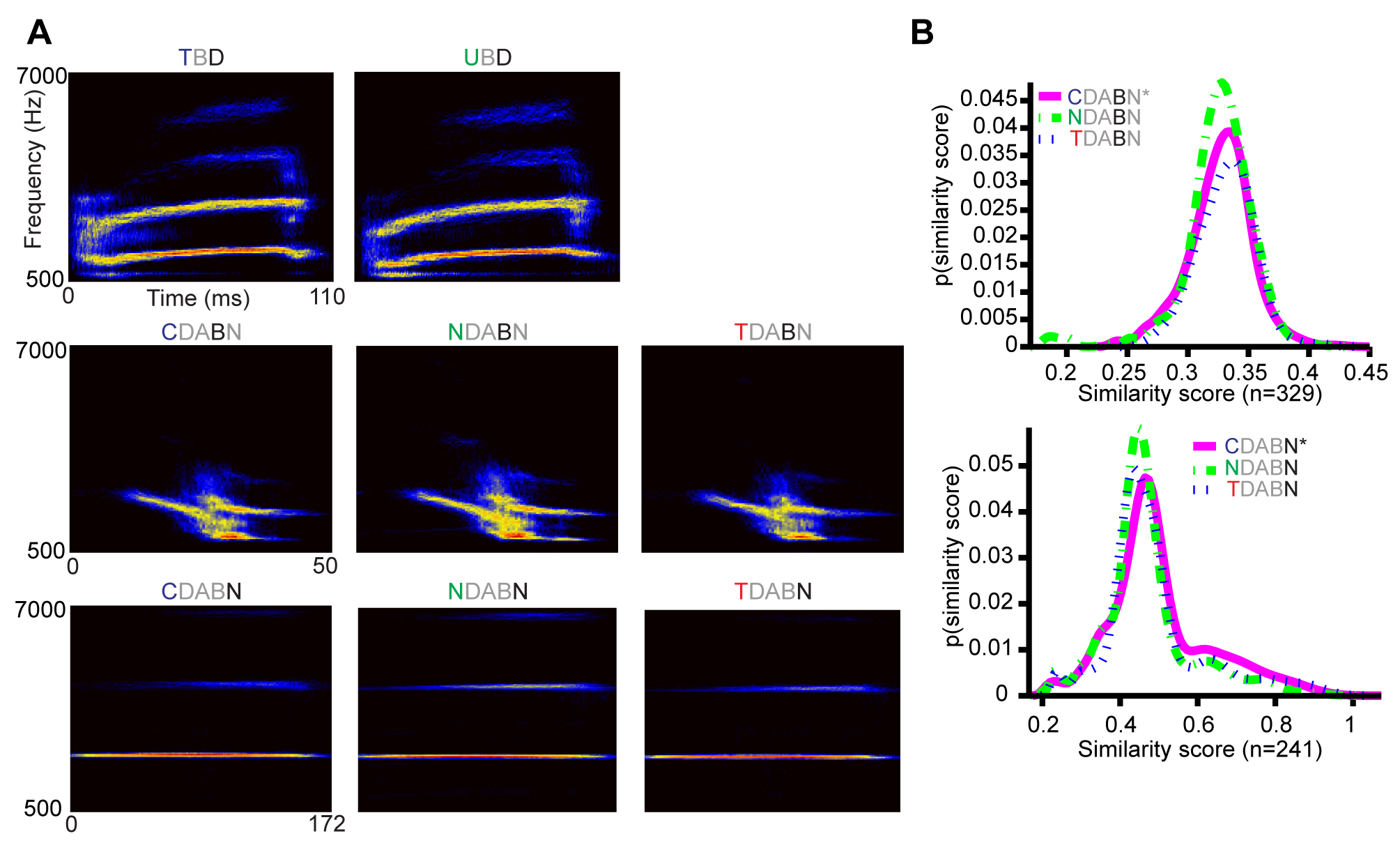

Supplement: Figure S5 — Spectral density images demonstrate that syllables occurring in different contexts are highly similar. A, Spectral density images of syllables with different surrounding phrases reveal acoustic similarity. As in Fig. 3a , spectral density images were computed for matching syllables that occur in different contexts; that is, different surrounding phrase types of earlier or later phrases. The particular phrase shown is highlighted in bold just above the image. The images were taken from 2 different birds. As in Fig. 3a , each row contains the same syllable type in three context groups that are acoustically indistinguishable by our statistical test. B, With the spectral density image from the magenta group as a reference, syllables occurring in different sequences have overlapping similarity score distributions. The syllable types match those shown in Fig. S5a. Summary statistics for all syllables types in Fig. S5a are given in Table S3. (TIFF) [file pcbi.1003052.s007.tiff]

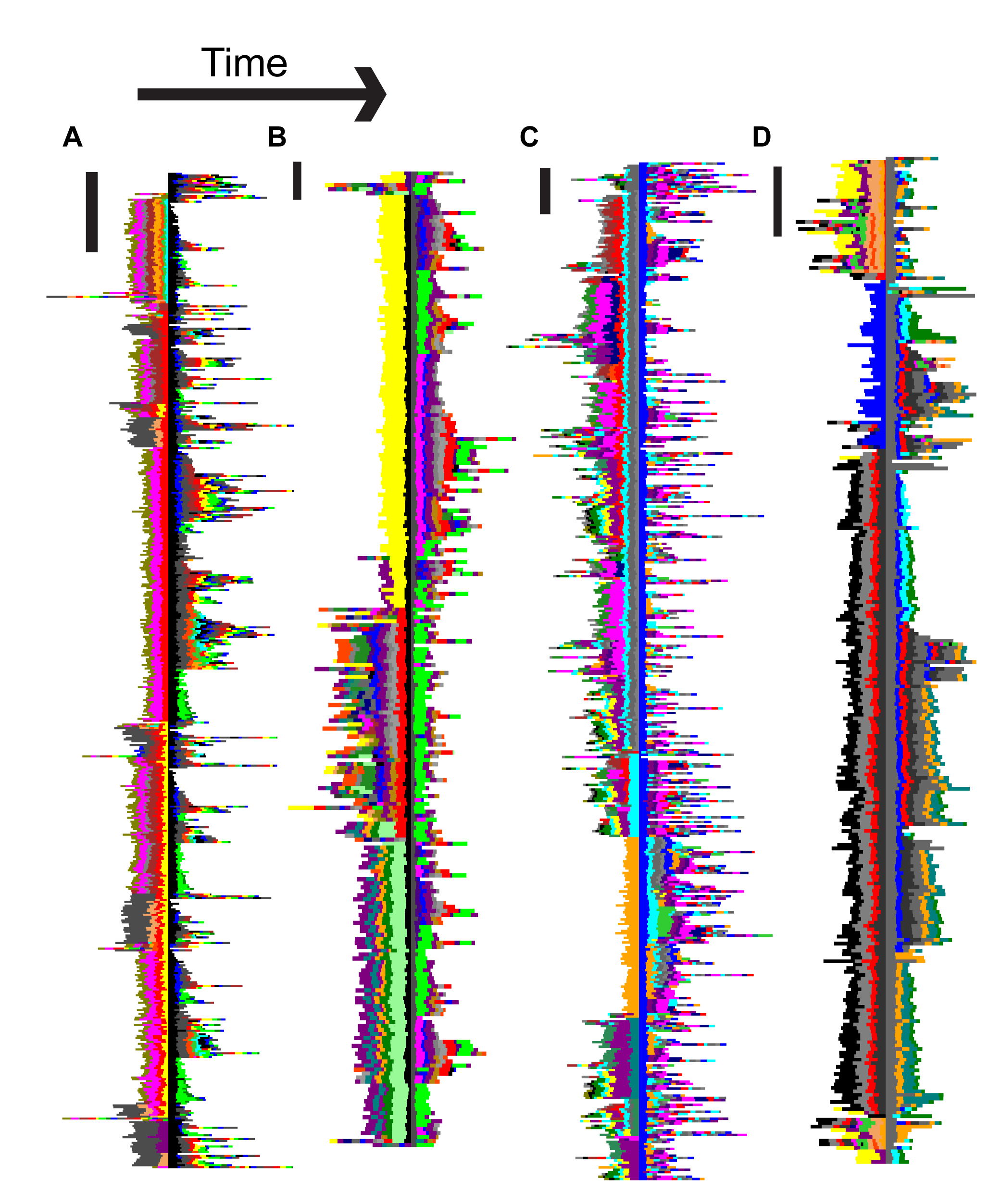

Supplement: Figure S6 — Full song barcodes for the examples given in Fig. 1 and Fig. 7 allow for direct visualization of long-range rules. These barcodes represent the full song sequence corresponding to the examples given in Fig. 1 and Fig. 7 . A, Barcodes for the example from Fig. 1a centered on the first occurrence of the black phrase. B, Barcodes for the example in Fig. 1b centered on the black and gray phrases. C, Barcodes for the example from the middle of Fig. 7 centered on the first occurrence of N (shown here in blue). D, Barcodes for the example from the bottom of Fig. 7 centered on the first occurrence of X. All colors match the phrases used in the corresponding parts of Fig. 1 and Fig. 7 , all other colors are arbitrarily assigned. A square flanks each barcode to indicate the scale, with the width corresponding to 2 seconds and the height to 20 trials. (TIFF) [file pcbi.1003052.s008.tiff]

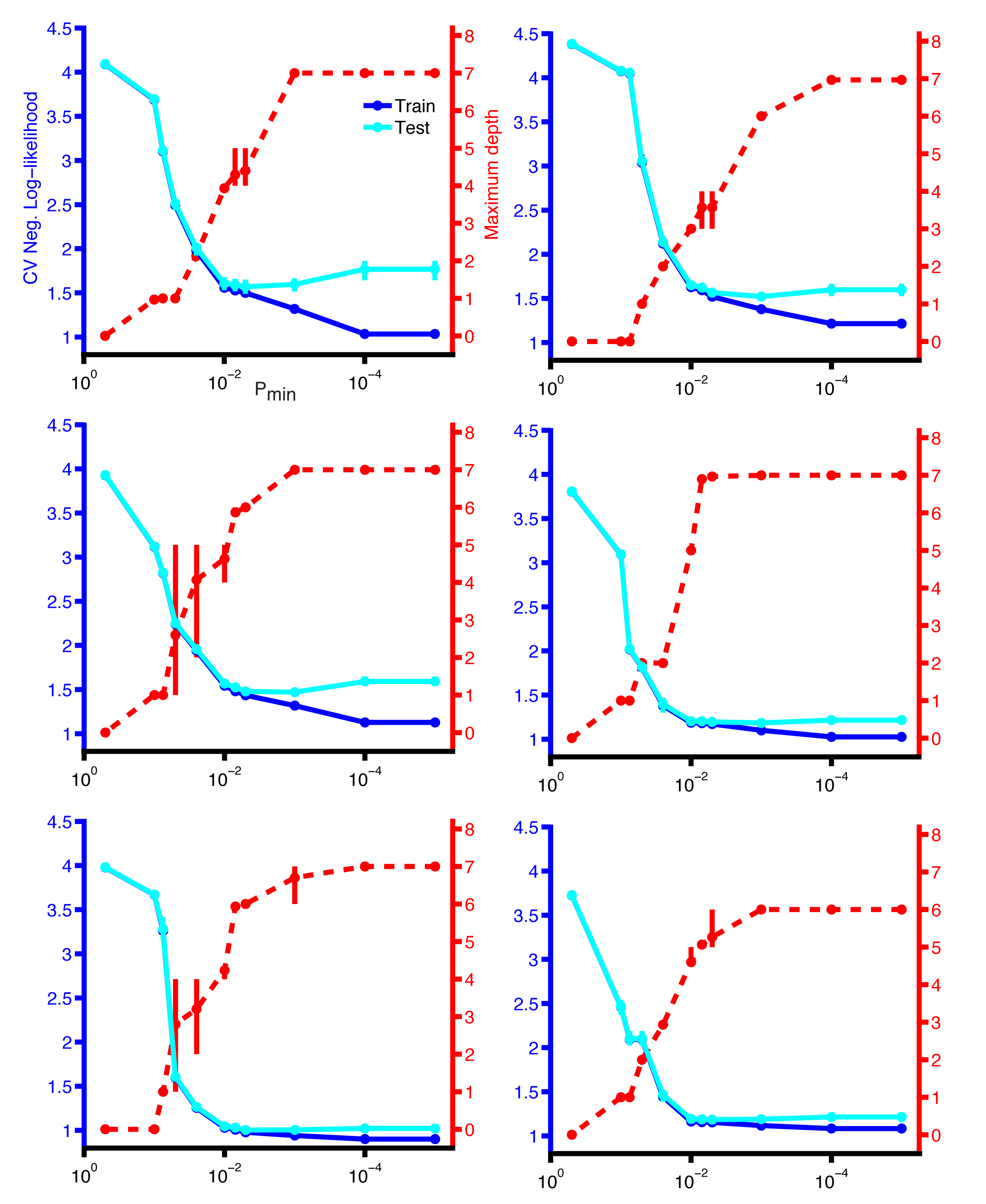

Supplement: Figure S7 — Using cross-validation to verify PST fits. To select the parameters used in the PST algorithm, we used a 10-fold cross-validation procedure repeated 3 times (with different data splits). As a measure of model performance we used average negative log-likelihood. Here we show cross-validation results as a function of the PST parameter . This parameter defines the minimum rate of occurrence for a sequence to be considered for incorporating into the PST. The plot shows the mean of the negative log-likelihood across cross-validation fits for the training and testing data for all 6 birds (error bars indicate the 25th and 75th percentiles). Also shown is the average maximum order of the PSTs for a given value of . Overfitting leads to a decline in performance on the test set, or increased negative log-likelihood. In each case, the optimal performance on the test set occurs when or . Below this point, performance on the test set degrades and sharply diverges from training set performance. We conservatively set to .007 for all birds to avoid overfitting. The same procedure was used to set and r, which had negligible effects on model performance (data not shown). (TIFF) [file pcbi.1003052.s009.tiff]

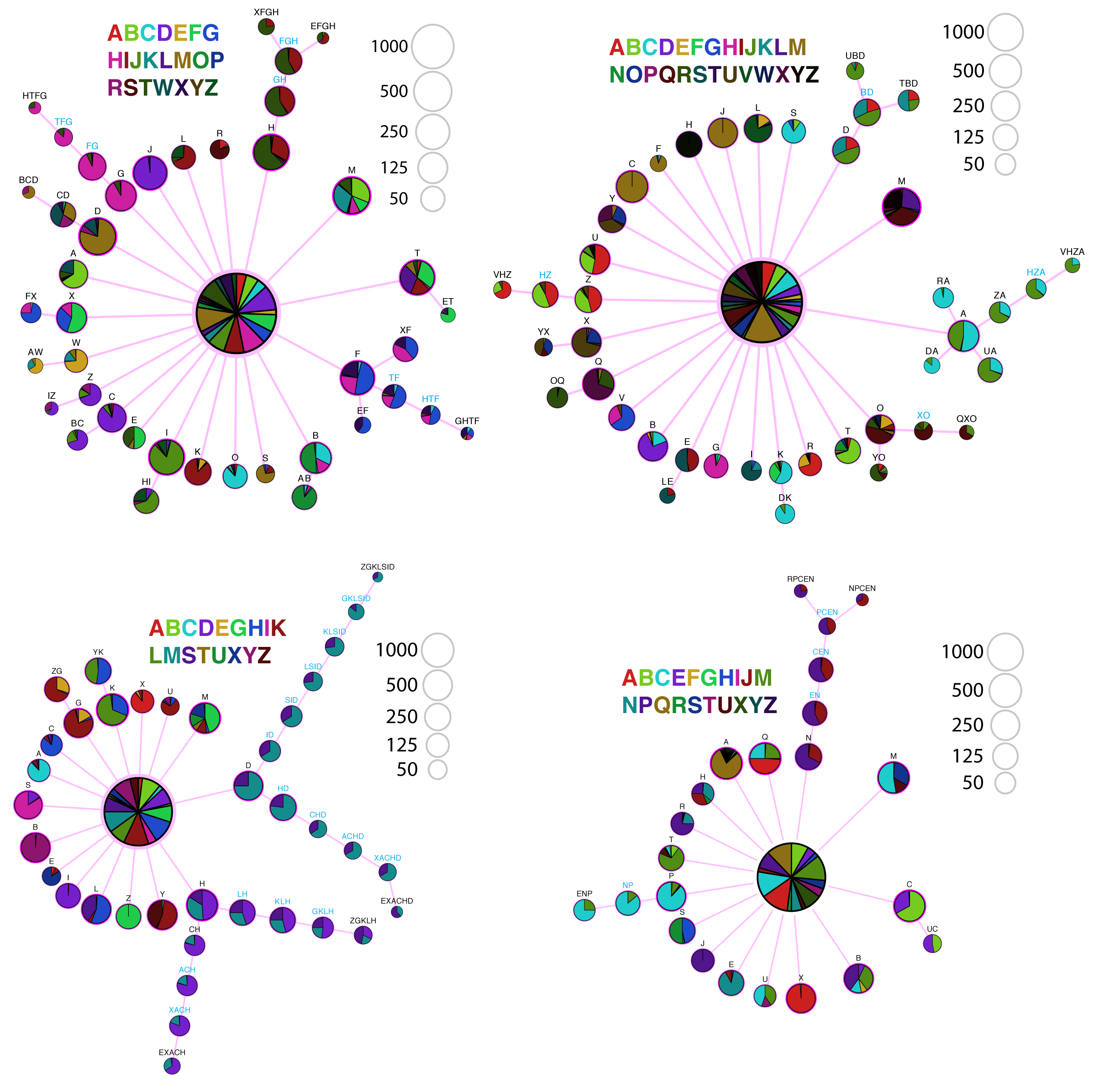

Supplement: Figure S8 — PSTs for the 4 birds not shown in Fig. 5 . (TIFF) [file pcbi.1003052.s010.tiff]

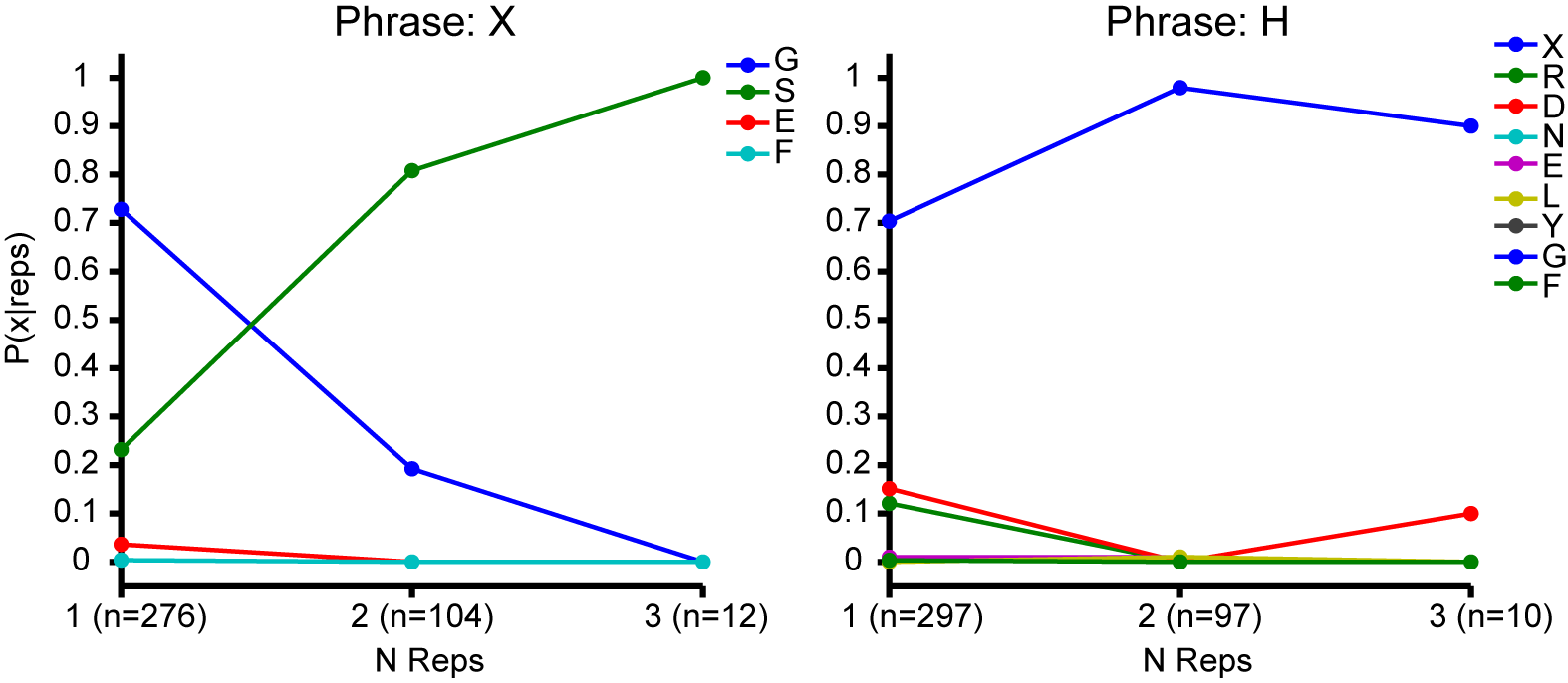

Supplement: Figure S9 — Shown are the transition probabilities from two different phrases sung by the same bird as a function of repetition number. Left: as phrase X is repeated, the most probable phrase transition from X to G decays, while the transition to S increases. Right: the opposite effect is seen in the same bird. The most probable transition, from H to X, increases as H is repeated. (TIFF) [file pcbi.1003052.s011.tiff]

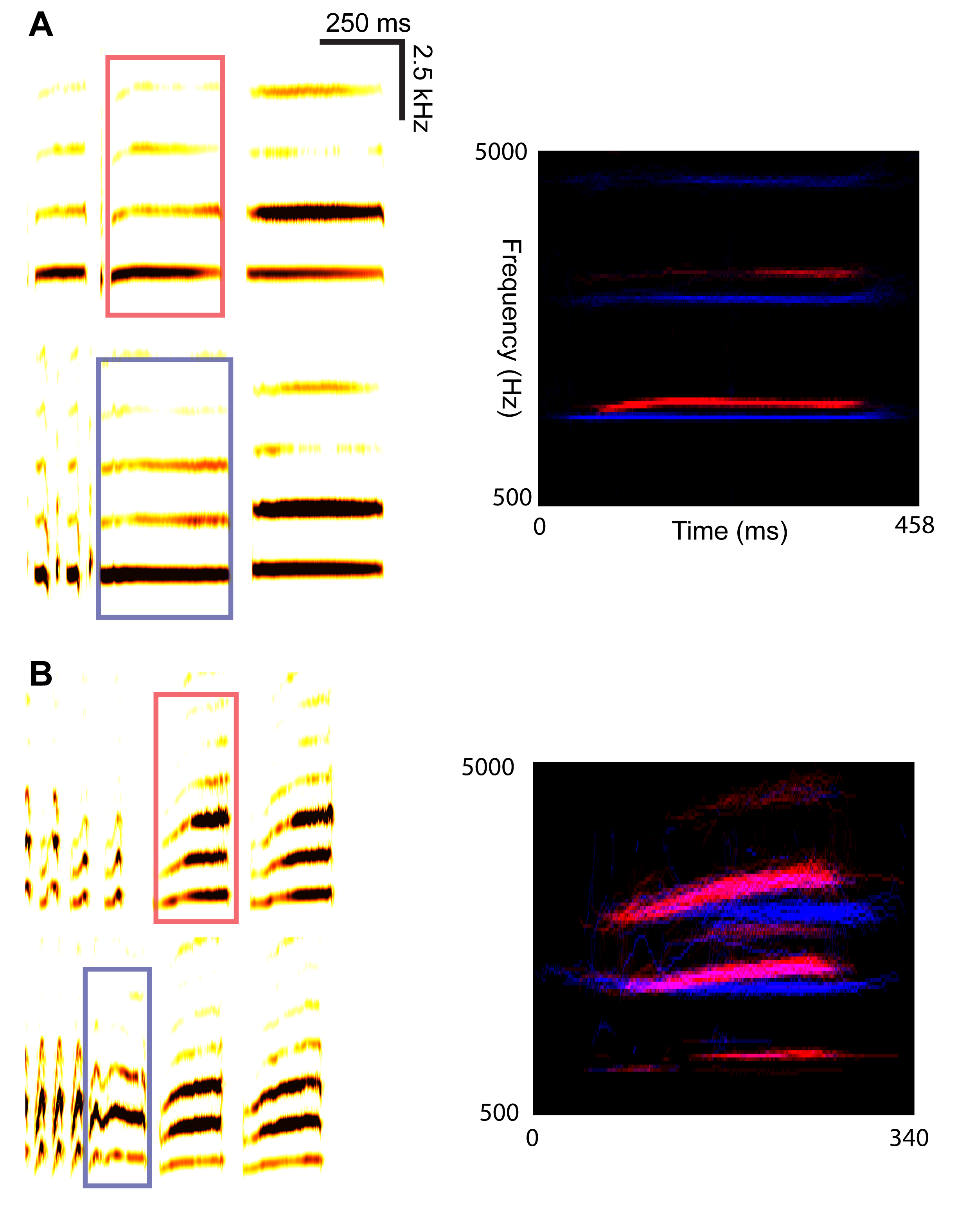

Supplement: Figure S10 — Some syllables have context-dependent transitional forms. A, shown on the left are two example sonograms of the same phrase with different preceding phrases. In the two contexts, the first syllable of the phrase has a different transitional form (highlighted by the red and blue boxes). The image on the right is a color channel merge of two spectral density images, which were computing using the first syllable of all phrases in the two different contexts (top sonogram context is the red channel and bottom sonogram context the blue channel). B, the same effect is shown for a different phrase from a different bird. (TIFF) [file pcbi.1003052.s012.tiff]

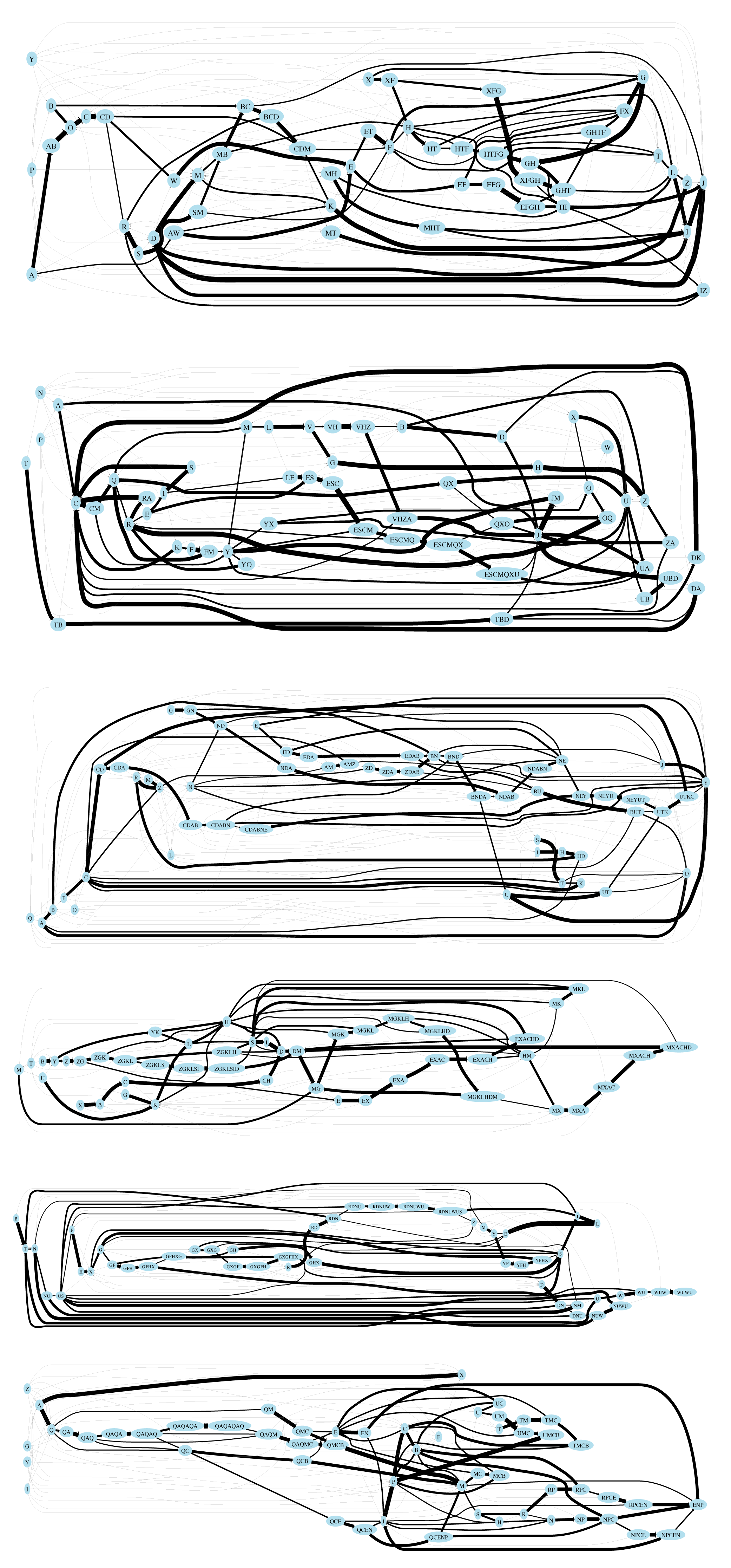

Supplement: Figure S11 — Probabilistic finite automata (PFA) for all 6 birds analyzed. For visualization, all edges where p<.05 have been removed, and all edges where p<.2 are shown in thin light gray lines. Each PFA is completely determined by its corresponding PST. From top to bottom, the PFAs correspond to the PST shown in: the top left of Fig. S8; top right of Fig. S8; top of Fig. 5 ; bottom left of Fig. S8; bottom of Fig. 5 ; bottom right of Fig. S8. (TIFF) [file pcbi.1003052.s013.tiff]

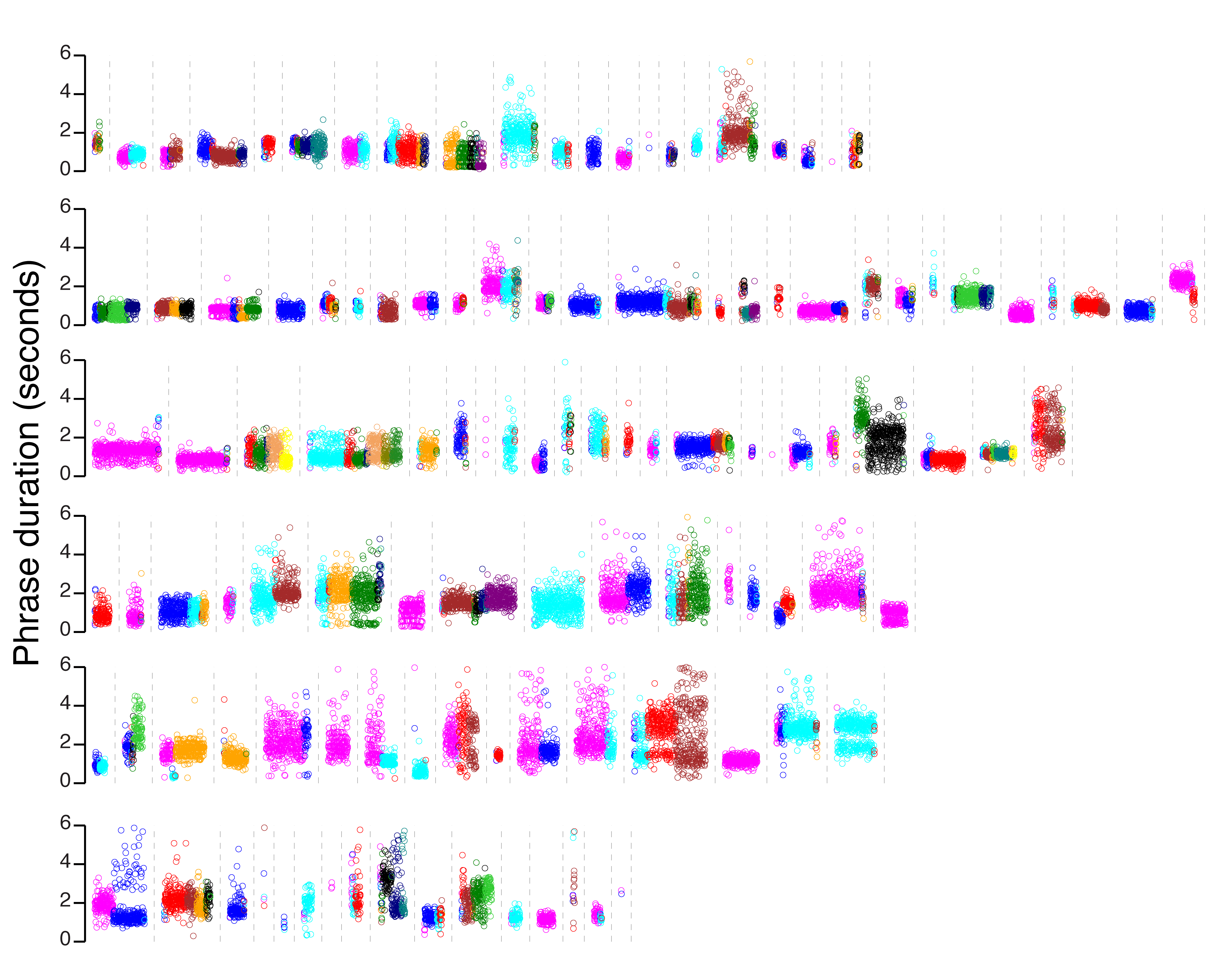

Supplement: Figure S12 — Phrase durations for all 6 birds analyzed. Each row corresponds to a different bird (same order as Fig. S11), and each group of points indicates the duration of a different phrase type. The colors (arbitrarily chosen) indicate different preceding phrase types. Abrupt changes in duration distribution that co-occur with color changes reveal a context-dependent shift in phrase length. (TIFF) [file pcbi.1003052.s014.tiff]
