# Supplementary material for: Long-range Order in Canary Song
Source: PLoS Comput Biol. 2013 May 2;9(5):e1003052. doi: 10.1371/journal.pcbi.1003052 (PMC3642045; doi:10.1371/journal.pcbi.1003052)
Supplement: Table S1 — The total number of phrases analyzed by each observer for individual birds. The bottom row contains the repertoire size for each bird. (DOCX) [file pcbi.1003052.s015.docx]

|  | Bird 1 | Bird 2 | Bird 3 | Bird 4 | Bird 5 | Bird 6 |
| --- | --- | --- | --- | --- | --- | --- |
| Observer 1 | 4606 | 6920 | 6264 | 5965 | 5832 | 3882 |
| Observer 2 | 5609 | 7175 | 6676 | 6580 | 6460 | 3947 |
| Repertoire | 22 | 26 | 22 | 17 | 18 | 20 |
