# Supplementary material for: Long-range Order in Canary Song
Source: PLoS Comput Biol. 2013 May 2;9(5):e1003052. doi: 10.1371/journal.pcbi.1003052 (PMC3642045; doi:10.1371/journal.pcbi.1003052)
Supplement: Table S2 — Summary statistics for similarity scores for duration groups (for each syllable type, scores were computed referenced to the spectral density image from the group marked*). STD, standard deviation. (DOCX) [file pcbi.1003052.s016.docx]

| *Syllable type* | *Duration group* | *N* | *Mean* | *STD* |
| --- | --- | --- | --- | --- |
| D | Short***** | 322 | .4756 | .0804 |
| D | Medium | 322 | .4591 | .0550 |
| D | Long | 322 | .4625 | .0620 |
| B | Short* | 1364 | .3553 | .0282 |
| B | Medium | 1364 | .3467 | .0257 |
| B | Long | 1364 | .3433 | .0317 |
| N | Short* | 1113 | .5037 | .1195 |
| N | Medium | 1113 | .4893 | .1118 |
| N | Long | 1113 | .4893 | .1010 |
