# Supplementary material for: Long-range Order in Canary Song
Source: PLoS Comput Biol. 2013 May 2;9(5):e1003052. doi: 10.1371/journal.pcbi.1003052 (PMC3642045; doi:10.1371/journal.pcbi.1003052)
Supplement: Table S3 — Summary statistics for similarity scores for sequence groups (for each syllable type, scores were computed referenced to the spectral density image from the group marked*). STD, standard deviation. (DOCX) [file pcbi.1003052.s017.docx]

| *Syllable type* | *Sequence group* | *N* | *Mean* | *STD* |
| --- | --- | --- | --- | --- |
| D | UB**D*** | 203 | .4306 | .0457 |
| D | TB**D** | 203 | .4351 | .0839 |
| B | CDA**B**N***** | 329 | .3266 | .0259 |
| B | NDA**B**N | 329 | .3250 | .0295 |
| B | TDA**B**N | 329 | .3308 | .0229 |
| N | CDAB**N*** | 241 | .5024 | .1311 |
| N | NDAB**N** | 241 | .4694 | .1147 |
| N | TDAB**N** | 241 | .4912 | .1296 |
