# Supplementary material for: Long-range Order in Canary Song
Source: PLoS Comput Biol. 2013 May 2;9(5):e1003052. doi: 10.1371/journal.pcbi.1003052 (PMC3642045; doi:10.1371/journal.pcbi.1003052)
Supplement: Table S4 — Transition probabilities and number of transitions to accompany Fig. 7 . The 95% confidence intervals are given in brackets below the transition probabilities and were estimated using a bootstrap procedure with a case resampling scheme. That is, the transition probabilities were estimated after randomly resampling the data with replacement. The confidence interval is then defined as the 2.5th and 97.5th percentiles of the resampled distribution. (DOCX) [file pcbi.1003052.s018.docx]

| Obs. | | Entry path | p(A\|*x*BD) | p(J\|*x*BD) | p(K\|*x*BD*)* |
| --- | --- | --- | --- | --- | --- |
| 1 | *x=*T | | .2308 (n=24)  [.1542 .3171] | .2596 (n=27)  [.1768 .3497] | .5096 (n=53)  [.4144 .6075] |
| 1 | *x=*U | | .0508 (n=3)  [0 .1132] | .8814 (n=52)  [.7863 .9623] | .0678 (n=4)  [.0160 .1379] |
| 2 | *x=*T | | .2288 (n=27)  [0 .3008] | .3051 (n=36)  [.2258 .3983] | .4492 (n=53)  [.3619 .5388] |
| 2 | *x=*U | | .0476 (n=3)  [0 .1111] | .8571 (n=54)  [.7675 .9375] | .0794 (n=5)  [.0172 .1478] |
|  |  | |  |  |  |
| Obs. | **Entry path** | | **p(D\|*x*DABN)** | **p(E\|*x*DABN)** | **p(Y\|*x*DABN)** |
| 1 | *x=*C | | .3577 (n=49)  [.2760 .4373] | .2044 (n=28)  [.1365 .2748] | .4380 (n=60)  [.3508 .5257] |
| 1 | *x=*N | | .1724 (n=5)  [.0417 .3182] | .6207 (n=18)  [.4422 .7971] | .1724 (n=5)  [.0417 .3218] |
| 1 | *x*=T | | .2368 (n=9)  [.0988 .3817] | .4737 (n=18)  [.3164 .6389] | .2895 (n=11)  [.1412 .4459] |
| 2 | *x=*C | | .3650 (n=50)  [.2794 .4465] | .1898 (n=26)  [.1245 .2599] | .4380 (n=60)  [.3608 .5192] |
| 2 | *x=*N | | .1481 (n=4)  [.0351 .2910] | .6667 (n=18)  [.4924 .8214] | .1852 (n=5)  [.0465 .3385] |
| 2 | *x*=T | | .2368 (n=9)  [.1062 .3817] | .4737 (n=18)  [.3164 .6364] | .2895 (n=11)  [.1515 .4417] |
|  |  | |  |  |  |
| Obs. | **Entry path** | | **p(E\|*x*HX)** | **p(G\|*x*HX)** | **p(S\|*x*HX)** |
| 1 | *x=*F | | .0038 (n=1)  [0 .0124] | .6038 (n=160)  [.56 .6533] | .3925 (n=104)  [.3423 .4346] |
| 1 | *x=*G | | 0  [0 0] | 0  [0 0] | 1 (n=35)  [1 1] |
| 2 | *x=*F | | .0036 (n=1)  [0 .0123] | .6051 (n=167)  [.5599 .6498] | .3877 (n=107)  [.3427 .4333] |
| 2 | *x=*G | | 0  [0 0] | 0  [0 0] | .9737 (n=37)  [.9091 1] |
