# Supplementary material for: Long-range Order in Canary Song
Source: PLoS Comput Biol. 2013 May 2;9(5):e1003052. doi: 10.1371/journal.pcbi.1003052 (PMC3642045; doi:10.1371/journal.pcbi.1003052)
Supplement: Table S5 — Transition probabilities demonstrating alternative paths for prediction suffix trees (PSTs) shown in Fig. 5 and Fig. S8. The sequence DABN comes from the top PST in Fig. 5 , HX from the bottom PST in Fig. 5 , and ZGKLH from the bottom left PST in Fig. S8. (DOCX) [file pcbi.1003052.s019.docx]

| Obs. | Path | n | p(A\|*x*) | p(B\|*x*) | p(N\|*x*) | p(R\|*x*) | p(U\|*x*) |
| --- | --- | --- | --- | --- | --- | --- | --- |
| 1 | *x=*DABN | 366 | 0 | 0 | 0 | 0 | 0 |
| 2 | *x=*DABN | 358 | 0 | 0 | 0 | 0 | 0 |
| 1 | *x=*DAB | 503 | 0 | 0 | .8 | 0 | .13 |
| 2 | *x=*DAB | 488 | 0 | 0 | .8 | 0 | .13 |
| 1 | *x=*DA | 683 | 0 | .77 | .02 | .04 | .04 |
| 2 | *x=*DA | 648 | 0 | .78 | .01 | .05 | .04 |
| 1 | *x=*D | 1039 | .71 | 0 | .04 | .15 | 0 |
| 2 | *x=*D | 969 | .72 | 0 | .04 | .16 | 0 |
|  |  |  |  |  |  |  |  |
| Obs. | **Path** | **n** | **p(D\|*x)*** | **p(E\|*x*)** | **p(G\|*x*)** | **p(R\|*x*)** | **p(S\|*x*)** |
| 1 | *x=*HX | 327 | 0 | 0 | .53 | 0 | .46 |
| 2 | *x=*HX | 313 | 0 | 0 | .53 | 0 | .46 |
| 1 | *x=*H | 472 | .09 | .04 | 0 | .09 | 0 |
| 2 | *x=*H | 437 | .11 | .01 | 0 | .09 | 0 |
|  |  |  |  |  |  |  |  |
| Obs. | **Path** | **n** | **p(D\|*x*)** | **p(E\|*x*)** | **p(K\|*x*)** | **p(M\|*x*)** | **p(S\|*x*)** |
| 1 | *x=*ZGKLH | 79 | .38 | 0 | 0 | .19 | .43 |
| 2 | *x=*ZGKLH | 67 | .32 | 0 | 0 | .21 | .47 |
| 1 | *x=*ZGKL | 136 | 0 | 0 | .02 | 0 | .37 |
| 2 | *x=*ZGKL | 120 | 0 | 0 | .03 | 0 | .39 |
| 1 | *x=*ZGK | 169 | .01 | 0 | 0 | 0 | .01 |
| 2 | *x=*ZGK | 154 | .01 | 0 | 0 | 0 | .01 |
| 1 | *x=*ZG | 254 | 0 | .30 | .67 | 0 | 0 |
| 2 | *x=*ZG | 227 | 0 | .30 | .68 | 0 | 0 |
| 1 | *x=*Z | 254 | 0 | 0 | 0 | 0 | 0 |
| 2 | *x=*Z | 228 | 0 | 0 | 0 | 0 | 0 |
