# Supplementary material for: Long-range Order in Canary Song
Source: PLoS Comput Biol. 2013 May 2;9(5):e1003052. doi: 10.1371/journal.pcbi.1003052 (PMC3642045; doi:10.1371/journal.pcbi.1003052)
Supplement: Text S1 — Supplementary Materials and Methods . (DOCX) [file pcbi.1003052.s020.docx]

**Text S1**

**Automated syllable clustering and alignment**

After choosing a template, the features from the template and the rest of the data were computed from a ratio of two quantities: the standard sonogram $(\chi)$ of the sound pressure time series $x(t)$, computed with a Gaussian window of time-scale $\sigma=1.5$ms, and a sonogram computed with the derivative of the Gaussian window $(\eta)$[1],

The complex phase of the ratio $\eta/\chi$ provides an exact measure of the direction of maximal spectral derivative. This direction of maximum spectral derivative has been employed previously in the characterization of zebra finch song, with slightly different mathematical definitions [2]. From these terms, we calculated spectral features: the local power in the sonogram $\left|\chi\right|$, , and a measure of how quickly the local spectral derivative is changing in time and frequency . Peaks in the cross-correlation over these features between the template and the data defined potential renditions of the template. As a final step, candidate sounds were plotted in two dimensions manually selected from the set of features defined above, and a decision boundary was drawn by the user.

**Inter-observer correlations**

To ensure that the phrase labeling was consistent, we first checked for qualitative similarity in the proportion of p-values for the Fisher-Freeman-Halton test computed from each set of labels (**Fig. S1**). Then to quantify the similarity, we looked at the correlation in test statistic values from each dataset for the same syllable type (**Fig. S2-4**).

**Prediction Suffix Trees**

Prediction suffix trees (PSTs) have previously been used for a variety of applications including data compression [3] and protein family prediction [4] but not yet applied to the analysis of song syntax. A suffix tree can be thought of as a set of variable order Markov chains—one chain for each element—in this case, one chain for every phrase type in the canary song. The algorithm to construct PSTs examines all strings up to length L that end in a particular syllable type. Starting with a zero-order Markov assumption, the algorithm then examines whether looking further into the past adds predictive information about the future sequence of the song. If looking further into the past for a syllable type adds information about the likely outcomes after singing the syllable, then the order of the Markov chain for that syllable type is increased. The algorithm is constructed to check all possible combinations and orders (up to a set maximum to save computation time).

The algorithm uses four parameters chosen by the user:

1) L, the maximum Markov order to check. Higher orders are not calculated for computational efficiency.

2) $p_{min}$ , a probability dictating that rare strings are disregarded. For example, to compare the transition probabilities from sequence XY against those from Y alone, $p(XY)$must exceed $p_{min}$.

3) $g_{min}$ and , which together specify a probability dictating that rare transitions are ignored. For example, if sequence XY passes condition 2, then it will be evaluated as part of the suffix tree if, for a given syllable Z . $g_{min}$ also specifies the amount of smoothing applied to transition probabilities per [5].

4) r, a quantity that defines the minimum information gain required to increase the Markov chain order. Specifically, it must be true that or , to justify adding XY to the suffix tree rather than terminating with Y.

We used a 10 fold cross-validation procedure to ensure that the PSTs were not overfitting the data. For each bird, we randomly split the data into 10 partitions, keeping 9 for training and using the 10^th^ for testing. We repeated the procedure 3 times and assessed PST performance using average negative log likelihood over both the training and test sets. Overfitting was characterized both by a degradation in test set performance, and by a divergence in performance between the training and test sets. The parameter with the most dramatic effect on model performance was $p_{min}$. The same parameters were used across all birds: , , , , and .

**Song Duration Effects**

For the examples given in **Fig. 1** and **Fig. 7** we found no evidence for song duration explaining context-dependence. The mutual dependence between phrase duration and the following syllable seen in **Fig. 1a** could be explained if the bird needed to stop after singing a long gray phrase, skipping from the blue phrase to the green phrase. The barcodes for the full song sequences (**Fig. S6a**) do not bear this out. The green phrase can follow a long gray phrase in a short bout that is less than half the duration of many other bouts. In another example, the phrase block in the center of **Fig. 1b** (dark and light gray) can be followed by the magenta, brown or green phrase. If the red phrase precedes the block green usually follows, whereas magenta usually follows when yellow precedes the block. Song duration cannot explain this long-range dependency since the green or magenta phrase can follow the block in the middle of a relatively short bout, or near the end of a much longer bout (**Fig. S6b**). Full song barcodes for the other examples in **Fig. 7** (**Fig. S6c** and **Fig. S6d**) show that song duration cannot explain these long-range dependencies either.

**Sound Analysis**

To validate the similarity score test, we compared acoustic features for the phrase groups shown in **Fig. 3** and **Fig. S5** using Sound Analysis Pro for MATLAB [2]. For each syllable in each phrase group we computed the amplitude modulation (AM), frequency modulation (FM), amplitude, entropy and the gravity center of the frequencies in a sliding 1 ms window. Then, each feature vector was normalized to z-scores. A pairwise distance matrix between all syllables was then computed using the Euclidean distance between the normalized features. (The syllables are aligned in the earlier extraction step, so the distance measure is computed for aligned time points, then summed over all time points in the syllable. )

The differences between two groups of syllables is computed as the D-prime separation between the self-similarity and cross-similarity scores. That is, the mean similarity for syllables within a group compared with the mean similarity for pairs of syllables drawn from different groups. This similarity is reported in units of standard deviation.

In all cases we found d’<.1.

**References**

1. Gardner TJ, Magnasco MO (2006) Sparse time-frequency representations. Proceedings of the National Academy of Sciences of the United States of America 103: 6094–6099. doi:10.1073/pnas.0601707103.

2. Tchernichovski O, Nottebohm F, Ho C, Pesaran B, Mitra P (2000) A procedure for an automated measurement of song similarity. Anim Behav 59: 1167–1176. doi:10.1006/anbe.1999.1416.

3. Begleiter R, El-Yaniv R, Yona G (2004) On prediction using variable order Markov models. J Artif Intell Res 22: 385–421.

4. Bejerano G, Yona G (2001) Variations on probabilistic suffix trees: statistical modeling and prediction of protein families. Bioinformatics 17: 23–43.

5. Ron D, Singer Y, Tishby N (1996) The power of amnesia: Learning probabilistic automata with variable memory length. Machine Learning 25: 117–149. doi:10.1023/A:1026490906255.
